# Human mesenchymal stem/stromal cell based-therapy in diabetes mellitus: experimental and clinical perspectives

**DOI:** 10.1186/s13287-024-03974-z

**Published:** 2024-10-29

**Authors:** Alaa Zeinhom, Sahar A. Fadallah, Marwa Mahmoud

**Affiliations:** 1 Biotechnology Department, Faculty of Science, Cairo University, Cairo Governorate, 12316 Egypt; 2 Human Medical Molecular Genetics Department, Human Genetics and Genome Research Institute, National Research Centre (NRC), Cairo, 12622 Egypt; 3 Stem Cell Research Unit, Medical Research Centre of Excellence, NRC, Cairo, Egypt

**Keywords:** Human mesenchymal stem/stromal cells, Diabetes, Experimental, Clinical, Beta-cell, Efficacy

## Abstract

Diabetes mellitus (DM), a chronic metabolic disease, poses a significant global health challenge, with current treatments often fail to prevent the long-term disease complications. Mesenchymal stem/stromal cells (MSCs) are, adult progenitors, able to repair injured tissues, exhibiting regenerative effects and immunoregulatory and anti-inflammatory responses, so they have been emerged as a promising therapeutic approach in many immune-related and inflammatory diseases. This review summarizes the therapeutic mechanisms and outcomes of MSCs, derived from different human tissue sources (hMSCs), in the context of DM type 1 and type 2. Animal model studies and clinical trials indicate that hMSCs can facilitate pleiotropic actions in the diabetic milieu for improved metabolic indices. In addition to modulating abnormally active immune system, hMSCs can ameliorate peripheral insulin resistance, halt beta-cell destruction, preserve residual beta-cell mass, promote beta-cell regeneration and insulin production, support islet grafts, and correct lipid metabolism. Moreover, hMSC-free derivatives, importantly extracellular vesicles, have shown potent experimental anti-diabetic efficacy. Moreover, the review discusses the diverse priming strategies that are introduced to enhance the preclinical anti-diabetic actions of hMSCs. Such strategies are recommended to restore the characteristics and functions of MSCs isolated from patients with DM for autologous implications. Finally, limitations and merits for the wide spread clinical applications of MSCs in DM such as the challenge of autologous versus allogeneic MSCs, the optimal MSC tissue source and administration route, the necessity of larger clinical trials for longer evaluation duration to assess safety concerns, are briefly presented.

**Graphical Abstract:**

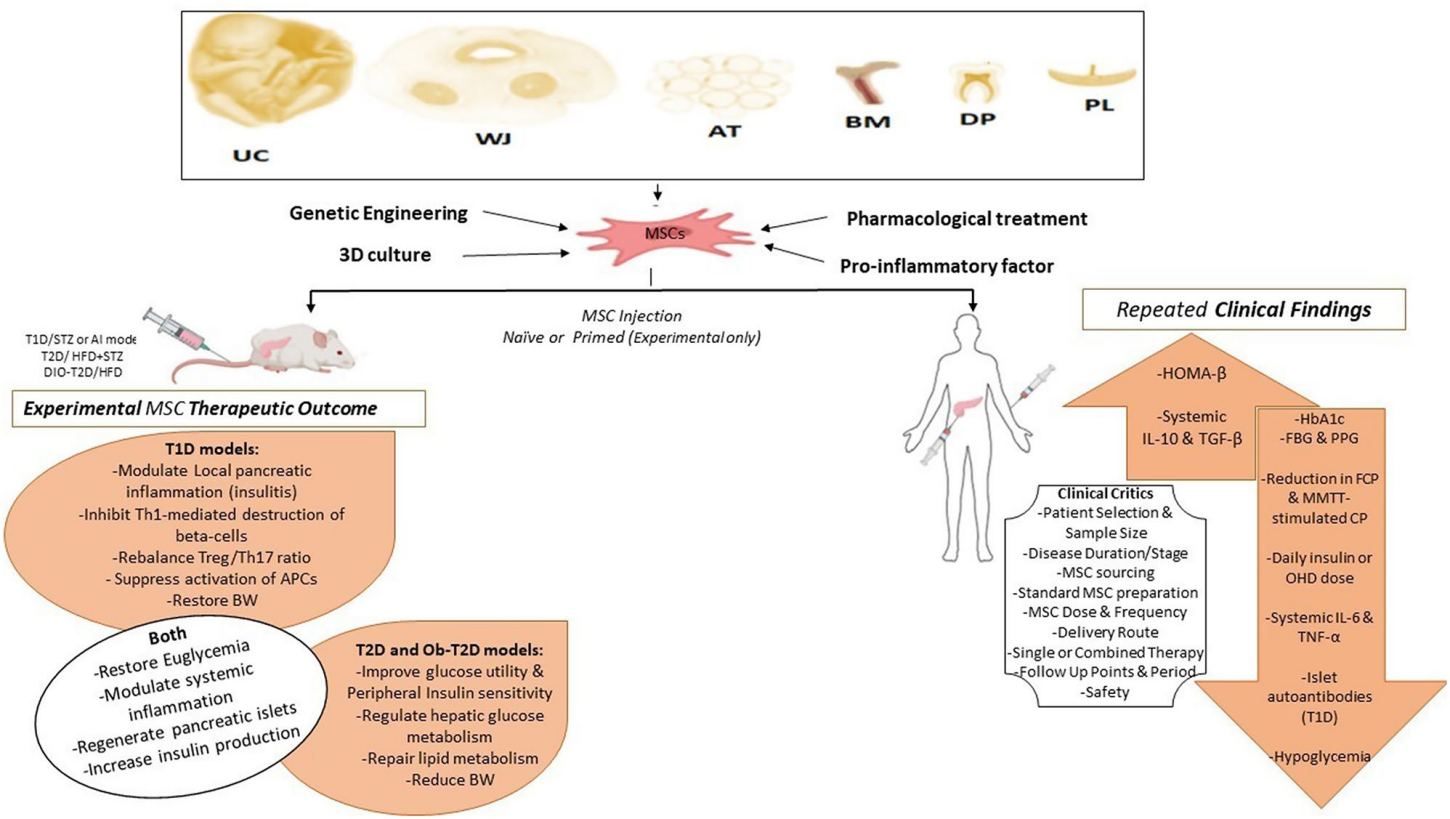

**Supplementary Information:**

The online version contains supplementary material available at 10.1186/s13287-024-03974-z.

## Introduction

Diabetes mellitus (DM) affects over 537 million people worldwide and represents a major health burden in industrial countries with estimated global direct health costs of more than 750 billion USD [1, 2]. DM is a metabolic disease that disturbs blood glucose level (BGL), it is classified into two major forms; type 1 diabetes (T1D) and type 2 diabetes (T2D). T1D is largely attributed to autoimmune attacks and genetic dysregulation against insulin producing beta-cells leading to insulin deficiency [3]. T2D accounts for 95% of diabetes cases. Patients with T2D are not sensitive to insulin and produce insufficient amounts of the hormone in the advanced disease stages [4, 5]. Obesity due to a Westernized high-calorie diet is considered the major cause of T2D [5]. Persistent hyperglycemia in patients with uncontrolled DM is associated with inflammation, oxidative stress and endoplasmic reticulum (ER) stress, leading to microvascular and macrovascular complications [6]. There are many conventional anti-diabetics, including oral drugs and exogenous insulin bolus, in addition to diet and exercise, that can temporarily reduce hyperglycemia or promote insulin sensitivity in target tissues. But unfortunately, they can’t reverse the disease development or cellular dysfunction. As well, severe hypoglycemia and poor adherence to treatment plans are limitations. Therefore, finding an effective long-term treatment for this disease is of highest priority [7, 8]. Pancreatic islet transplantation (PIT) holds great promise for treatment of insulin-dependent patients [9]. However, poor survival of isolated islets, immunological rejection, significant postoperative difficulties, and a scarcity of donors limit the wide spread application of PIT [7, 10].

In recent years, cell-based therapy using mesenchymal stem/stromal cells (MSCs) is of great interest for DM [11–13] MSCs have been successfully isolated from a variety of adult tissues, predominately from bone marrow (BM) [14], adipose tissue (AT) [15, 16], and dental pulp (DP) [17], or extraembryonic tissues such as placenta [18], umbilical cord (UC) [19] and amniotic fluid (AF) [20, 21]. In addition to fulfilling the three minimal criteria for MSC definition (plastic adherence, expression of a panel of surface markers, tri-mesodermal lineage differentiation), the International Society for Cell and Gene Therapy (ISCT) in 2019 recommended to demonstrate the functional properties of MSCs based on standardized functional assays, such as in vitro analyses of their trophic secretome, immunomodulatory properties, and angiogenic functions [22]. Noteworthy, MSCs from different sources barely express the major histocompatibility molecules class II and costimulatory molecules such as CD40, CD80, and CD86, thus they have been proposed as hypoimmunogenic cells [23, 24], however, MSC immuneprivileged behavior is environmental context-dependent, so it is not consistent [25].

### Therapeutic mechanisms of MSCs in DM

**Fig. 1:**
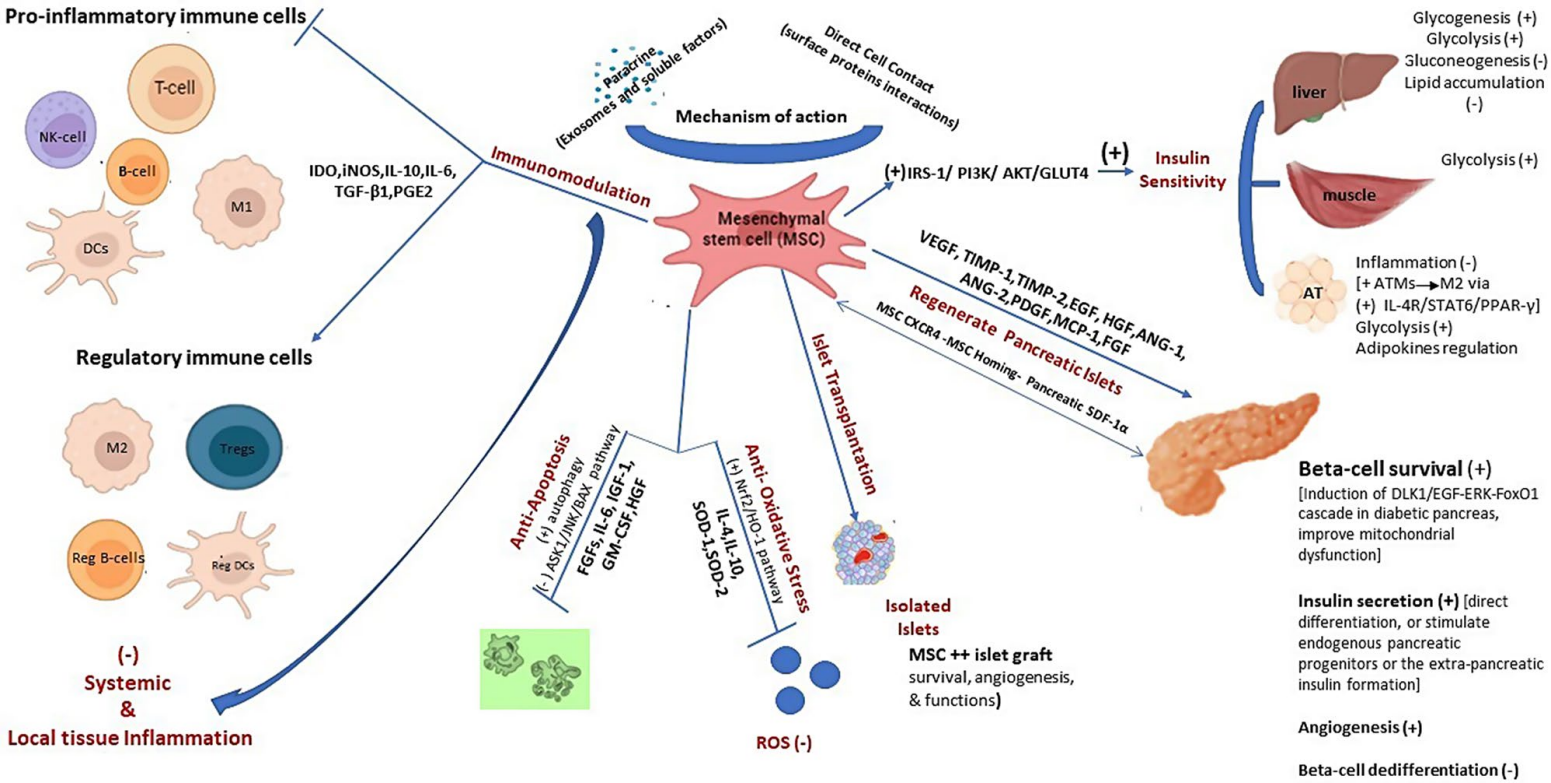
Therapeutic Mechanisms of MSCs in DM management. *Abbreviations* *ANG-1* Angiopoietin 1, *ANG-2* Angiopoietin 2, *AT* Adipose tissue, *CXCR4* C-X-C chemokine receptor type 4, *DCs* Dendritic cells, *DLK1* Delta like non-canonical Notch ligand 1, *EGF* Epidermal growth factor, *ERK* Extracellular signal-regulated kinase, *FGF* Fibroblast growth factor, *FoxO1* Forkhead box protein O 1, *GLUT4* Glucose transporter 4, *GM-CSF* Granulocyte macrophage colony stimulating factor, *HGF* Hepatocyte growth factor, *HO-1* Heme oxygenase 1, *IDO* Indoleamine 2,3-dioxygenase, *IL-10* Interleukin 10, *IL-4* Interleukin 4, *IL-6* Interleukin 6, *IGF-1* Insulin-like-growth factor, *iNOS* Inducible-nitric oxide synthase, *IRS-1* Insulin receptor substrate 1, *M1* Pro-inflammatory macrophages, *M2* Anti-inflammatory macrophages, *MCP-1* monocyte-chemotactic protein 1, *MC4R* melanocortin-4 receptor, *NK-cells* Natural killer cells, *Nrf2* Nuclear factor erythroid 2-related factor, *PDGF* Platelet-derived growth factor, *PGE2* Prostaglandin E2, *Reg B cells* regulatory B cells, *Reg DCs* Regulatory dendritic cells, *ROS* Reactive oxygen species, *SDF-1α* stromal-derived factor 1 alpha, *SOD-1* Superoxide dismutase 1, *SOD-2* Superoxide dismutase 2, *TIMP-1* Tissue inhibitor of metalloproteinase 1, *TIMP-2* Tissue inhibitor of metalloproteinase 2, *TGF-β1* Transforming growth factor beta 1, *Tregs* Regulatory T cells, *VEGF* Vascular endothelial growth factor, (+): promote/support, (-): inhibit or suppress

Human tissue-derived MSCs (hMSCs) have been validated in treating different degenerative [26], inflammatory [27], or autoimmune [28], diseases. In DM, hMSCs exhibit multifaceted therapeutic actions (Fig. 1). Depending on their well-established immunoregulatory ability, hMSCs can modulate various kinds of innate and adaptive immune cells in inflammation [29]. The MSC immunomodulatory effects include, among others, the inhibition of autoreactive T cells’ proliferation and activation, thereby halting the destruction of pancreatic beta-cells in T1D [30]. Moreover, they can promote the generation and/or expansion of regulatory T cells (Tregs), crucial for maintaining immune tolerance, to prevent autoimmune attacks on pancreatic beta-cells [31–33]. MSCs promote the M2 macrophages for the favor of improved peripheral insulin sensitivity in T2D [34, 35]. M2 macrophages are considered to be critical effector cells in the resolution of inflammation and the promotion of tissue repair [36]. MSCs exert the immunomoregulatory functions via surface proteins-mediated direct interaction with immune cells or paracrine mechanisms [37]. The paracrine function includes the secretion, in response to inflammation, of extracellular vesicles or the release of anti-inflammatory molecules such as indoleamine 2,3-dioxygenase (IDO), interleukin-10 (IL-10), transforming growth factor-beta (TGF-β), interleukin 6 (IL-6), interleukin 1 receptor antagonist (IL-1RA), tumor necrosis factor-stimulated gene 6 (TSG-6), and prostaglandin E2 (PGE2) [32, 34, 38].

Importantly, hMSC- mitigate insulin resistance (IR) leading to enhanced glucose uptake by peripheral tissues such as skeletal muscle, liver, and AT, thereby restoring glycemic control, promoting beta-cell recovery, and reducing the risk of T2D-related complications [39, 40]. MSCs ameliorate peripheral tissues IR via phosphatidyl inositol tri-kinase (PI3K)-enhanced phosphorylation of insulin-receptor substrate 1 (IRS-1) which in turns upregulates glucose transporter 4 (Glut4) and insulin receptor expression on cell membrane [41–43], and via downregulation of stress-induced serine kinases, such as c-Jun terminal kinase 1 (JNK1) and extracellular-regulated kinase 1 (ERK1) [44]. Moreover, MSCs attenuate high glucose-induced oxidative stress in beta-cells via the nuclear factor erythroid 2-related factor/ heme oxygenase 1 (Nrf2/HO-1) signaling pathway [32, 45], and regulate hepatic glucose [46, 47], and glycolipid [48] metabolism. Additionally, hMSCs reduce intracellular reactive oxygen species (ROS) levels in diabetic beta-cells by at least partially autophagy mediated-improving of mitochondrial functions [49].

MSCs have the ability to promote insulin production via different mechanisms. hMSCs from different sources have been shown to differentiate into glucose-responsive insulin-producing beta-cells (IPCs) in vitro [13, 50–54], and in vivo [51, 55–57]. Moreover, MSCs enhance the pancreatic microenvironment and restore beta-cell function by at least partially preventing beta-cell apoptosis [57], and supporting their survival [58]. The combined therapy of human UC derived-MSCs (hUC-MSCs) and liraglutide, a glucagon-like-peptide 1 (GLP1), additively inhibited beta-cell apoptosis in T2D model via suppressing the ASK1/JNK/BAX signaling [59]. From another perspective, hMSCs secrete trophic mediators and growth factors, in response to injury, such as vascular endothelial growth factor (VEGF) fibroblast growth factor (FGF), angiopoietin-1, and hepatocyte growth factor (HGF) that promote angiogenesis, enhancing blood flow and nutrient delivery to beta-cells [57, 58]. Moreover, studies in T2D patients [60], and experimental models [61–63] indicate that beta-cell dysfunction in hyperglycemia is mainly due to beta-cell dedifferentiation (loss of canonical beta-cell markers and regression to an endocrine progenitor-like stage). Regulation of glucose and lipid metabolism contribute to MSC reversal of beta-cell dedifferentiation in T2D model [62], and that has been reported to bean IL-1RA dependent [63]. Such findings support the reversal of beta-cell dedifferentiation as one the potential MSC therapeutic mechanisms in DM.

Notably, hMSCs play a significant role in promoting PIT [10, 58, 64–71]. hMSCs improve islet engraftment, survival, angiogenesis, and function [64, 70]. MSC-islet composite before transplantation has been suggested to promote the transplant immune tolerance in vivo [65]. Human MSCs derived from BM (hBMSCs) or AT (hASCs) cotransplanted with neonatal porcine islets in an extrahepatic site augmented the anti-diabetic effects of the islet xenograft in diabetic mice [68, 71]. In another report, coculture of hASCs with murine or human islets potentiated the islet graft viability and glucose-stimulated insulin release and interestingly it was a critic for restoring normoglycemia in diabetic mice, where, transplantation without coculture had a detrimental effect [69]. This effect could be due to the MSC abundant release of VEGF, interleukin 6 (IL-6), and/or tissue inhibitor of metalloproteinase 1 (TIMP-1) [58, 70], and by the reduction of the inflammatory markers; tumor necrosis factor alpha (TNF-α), interleukin 1 beta (IL-1β), and monocyte chemotactic protein 1 (MCP-1) in the graft [67, 70].

*In the coming review sections*, we discuss studies that illustrate the experimental potential of hMSCs from different sources in T1D and T2D animal models. The experimental anti-diabetic potential of MSC-free derivatives especially, exosomes, are also briefly summarized. We then present some strategies that are recommended to potentiate the anti-diabetic effect of hMSCs at preclinical level. Finally, the up-to-date clinical trials in the context of MSCs and diabetes are reviewed to conclude the clinical significance of hMSCs in DM metabolic abnormalities management.

### Preclinical evidence for the anti-T1D potential of undifferentiated hMSCs

Strong preclinical evidence for the therapeutic efficacy of hMSC transplantation in experimental T1D has been illustrated [32, 38, 41, 45, 51, 55, 56, 58, 72–85] (Table 1). In those studies, T1D was modelled by injecting consequent small doses or a single large dose of streptozotocin (STZ), which induces diabetes by disrupting of islet structure [86], then monitoring BGL to achieve stable high values ranging from 200 to 500 mg/dl. From day 7 up to 21 days of STZ injection, the model was established and MSC transplantation was performed. Alternatively, genetically modified non-obese diabetic (NOD) mice that develop spontaneous T-cell-dependent beta-cell destruction that resembles human T1D were used [86]. In those experimental T1D models, MSCs, from different sources, were administered, including mainly those derived from UC/WJ, then BM or AT, and the least frequency reported for those derived from dental tissues.

**Table 1:** Undifferentiated hMSC-based therapy in experimental T1D

Ref.	DM model Induction	MSC source	Cell dose & Frequency	Route of administration	Study Experimental Groups	End of Follow up	Main findings
51	Ten-week-old BALB/c mice were intraperitoneally injected with a single dose of STZ (200 mg/kg BW)	PL-MSCs Vs PL-MSC-derived ILCs	A single dose of (1.5 × 10^5^) of MSCs/mouse or approximately 1000 islet equivalent ILCs	Under the kidney capsule	Gp1: PL-MSC-treated Gp2: PL-MSC-ILC-treated	Almost 4 weeks	-Both undifferentiated PL-MSCs and their ILCs restored normoglycemia and GSIS in STZ-mice.
55	Ten-week-old BALB/c mice were intraperitoneally injected with a single dose of STZ (200 mg/kg BW)	BMSCs	Single injection (4.2 × 107 cells/kg) (7 weeks of STZ) vs. Multiple Injections (4.2 × 107 cells/kg each time at 2-weeks interval for 6 months) (1-week post-STZ)	Tail vein	Gp1: Single-dose MSC-treated Gp2: Multiple-doses MSC-treated Gp3: STZ mice + PBS Gp4: Healthy controls	24 weeks	-Compared to single transplantation, which transiently decreased hyperglycemia, multiple MSC transplantations effectively restored blood glucose homeostasis through inhibiting systemic oxidative stress from the 7th week of treatment and engraftment of the host liver and differentiation into IPCs from the 11th week, accounting for the long-term therapeutic effects of MSCs.
58	NOD/SCID/ gchain^null^ mice intra-peritoneally injected with 45 mg/kg STZ for 4 days.	ASCs	A single dose of 2 × 10^6^ cells /Animal	Tail vein	Gp1: hMSC treated Gp2: STZ mice injected with PBS only Gp3: Normal mice: No STZ, No MSC	5 weeks post- MSC transplant	- Enhanced glucose tolerance -Improved beta-cell mass & boosted beta-cell survival and proliferation via TIMP-1 dependent mechanism
72	Male mice (aged 6–8 weeks) intravenously administered a dose of 50 mg/kg STZ for 5 days	UC-MSCs	A single dose of 5 × 10^6^/mouse	IV or IP	Gp1: IV-UC-MSC-treated Gp2: IP-UC-MSC-treated Gp3: STZ mice treated with IV PBS Gp4: STZ mice treated with IP PBS Gp5: Normal mice: No STZ, No MSC	Almost 4 weeks	-Both administration routes decreased blood glucose concentrations, increased the serum insulin, and supported pancreas recovery in treated mice. -However, IV injected MSCs had higher recovery effect than those injected by IP.
74	Eight-week-old male NOD/ SCID mice were treated with 50 mg/kg BW STZ for 3 days	AF-MSCs	A single dose of 1 × 10^6^ MSCs/rat One day post STZ injection	Intracardiac	Gp1: MSC-treated Gp2: fibroblast-treated Gp3: disease model + NS Gp4: healthy mice + NS	4 weeks	-AF-MSC injection resulted in protection from beta-cell damage and increased beta-cell mass -beta-cell regeneration correlated with activation of the IRS-1 /PI3K/Akt signaling pathway and VEGFA expression
73	Rats administered a single dose of 70 mg/kg STZ	UC-MSCs	A single dose of 5 × 10^6^ MSCs/rat	Tail vein	Gp1: STZ + UC-MSCs Gp2: STZ + PBS	4 weeks	-Hyperglycemia and BW were improved -Pancreatic islet destruction was partially repaired
41	Female NOD mice with age six-weeks	WJ-MSCs	A single dose of 1 × 10^6^ cells/mouse Injected one week after spontaneous diabetes incidence	IV	Gp1: WJ-MSCs treatment group (after onset) Gp2: WJ-MSCs prevention group (before onset) Gp3: normal control group Gp4: diabetic control group	18 weeks	-FPG and fed BGL in WJ-MSCs treatment group decreased to normal level in 6–8 days and maintained for 6 weeks. -Level of fasting C-peptide of those mice was significantly higher compared to diabetic control mice. -WJ-MSCs played a protective role (delayed onset of diabetes for 8-weeks) in WJ-MSCs prevention group. -Compared with diabetic control group, frequencies of CD4^+^CD25^+^Foxp3+ Tregs in WJ-MSCs prevention and treatment groups were significantly higher, while levels of IL-2, IFN-γ, and TNF-α were lower. - The degree of insulitis was also depressed, especially for WJ-MSCs prevention group.
75	Female NOD mice aged twenty-weeks (with a spontaneous diabetes incidence)	WJ-MSCs	A single dose of 5 × 10^5^ MSCs/mouse	IV (retro-orbital vein)	Gp1: MSC-treated Gp2: diabetic control + NS	1 week vs. 3 weeks	-Undifferentiated WJ-MSCs differentiated into IPCs in vivo with immunomodulatory effects and repair the destroyed islets in NOD mice. Immunomodulatory effects represented in: • Systemic and local levels of auto aggressive T-cells, including Th1 cells and IL-17-producing T-cells, were reduced, and regulatory T-cell levels were increased. • Anti-inflammatory cytokine levels were increased, and DCs were decreased. -At the end of observation, higher human C-peptide and serum insulin levels and improved glucose tolerance were found. -Significantly more intact islets and less severe insulitis were observed.
32	Seven-eight weeks of age Female NOD/Ltj mice were injected intraperitoneally with 40 mg/kg STZ for 5 days	BMSCs	A single or two doses (1 week-interval) of the low dose; 0.5 × 10^6^ MSCs/animal A single dose of the high dose; 1.0 × 10^6^ MSCs/animal	Tail vein	Gp1: Low-dose MSC Gp2: high-dose MSC Gp3: multiple-dose MSC Gp4: T1D controls: Hank’s solution only Gp5: Healthy controls	6 weeks post- MSC transplant	-Increased plasma insulin level and islet insulin content. -Reduced pancreas insulitis -Decreased pancreatic TNF-α, and increased IL-10 and TGF-β1 expression. -Increased percentages of splenic Tregs and levels of plasma IL-4, IL-10 and TGF-β1, -Reduced percentages of splenic CD8^+^ T and levels of plasma IFN-γ, TNF-α and IL-17 A. -MSC localization in pancreas was detected by day 28 post-transplant -MSC beta-cell protective and immunoregulatory effects tended to be dose-dependent and multiple doses of MSCs held longer effects.
76	C57BL/6 male mice (ten-weeks of age) were intraperitoneally injected 40 mg/kg STZ for 5 consecutive days	BMSCs	A Single dose of 1 × 10^6^	IP	Gp1: C-MSC-treated (Diabetic mice treated with MSCs isolated from healthy individuals) Gp2: T1D-MSC-treated (Diabetic mice treated with MSCs isolated from patients with newly diagnosed T1D- Gp3: Control (Diabetic mice treated with PBS)	5 weeks Post-MSC transplant	-MSCs, even those isolated from diabetic patients, were able to: • Reverse hyperglycemia • Improve islet morphology and beta cell function (Increased insulin production) • Enhance beta-cell proliferation • Modulate pancreatic cytokine levels (significant reduction of IFN-γ and IL-2).
77	Eight-week-old male mice intraperitoneally injected with a single dose of 100 mg/kg STZ	ASCs	Two different doses were tested 1 × 10^6^ vs. 2 × 10^6^ MSC/mouse	IV	Gp1: High-dose MSC-treated Gp2: Low-dose MSC-treated Gp3: STZ mice injected with PBS only	8 weeks Post-MSC transplant	-High MSC dose, compared with the low one: • Decreased death rate • Induced better improvement in glucose levels and tolerance • Promoted better insulin production & release. • Restored islet architecture and prevented further destruction.
78	Male Kunming mice (ten-week-old) injected intraperitoneal of an initial dose of 180 mg/kg STZ, followed by 100 mg/kg for another 2 consecutive days	WJ-MSCs	A single dose of 1 × 10^7^ MSC/mouse 2–3 weeks post STZ	IP	Gp1: UC-MSC treated Gp2: control dibetic	11 weeks	- MSCs migrated to damaged tissues (liver, kidney, pancreas, spleen). -MSC reverted hyperglycemia and increased the serum mouse c-peptide -MSCs promoted insulin production from non-pancreatic local cells. -MSCs repaired STZ-induced renal damage
79	C57/BL6 mice administered STZ (115 mg/kg) by intraperitoneal injection	BMSCs	1 × 10^6^ cells (Day 7 post STZ injection) In a group a second IP injection of 10^6^ MSCs/animal was administered at day 28 post STZ	IP or IV	Gp1: IV hBMSCs Gp2: IP hBMSCs Gp3: IV vehicle Gp4: IP vehicle Gp5: IP-hBMSCs (two doses)	3 weeks post- MSC transplant	-IP, but not IV, hBMSC injection significantly reduced BGL on day 21 compared with vehicle injection by the same route. -IP injected fluorescence-labeled hBMSCs were observed in the intra- and extra-lobular spaces of the pancreas, and intravenously injected cells were in the lung region, although the number of cells mostly decreased within 2 weeks of injection. - Animals injected with IP- hBMSCs twice exhibited increases in the plasma insulin level, number and size of islets, insulin-positive proportion of the total pancreas area, and intensity of insulin staining compared with vehicle-injected animals. - Decrease of Iba1 (a pan macrophage marker)-positive cells in islets and an increase of CD206 (M2 macrophage marker)-positive cells in both the endocrine and exocrine pancreas. - The hBMSC injection also reduced the number of CD40-positive cells merged with glucagon immunoreactions (α-cells) in the islets.
57	Male SD rats (aged six-weeks) were Injected intraperitoneally with a single dose of 50 mg/kg STZ	DPSCs	A single dose of 1 × 10^6^ cells/rat (7 days post-STZ)	IV or IP	Gp1: IV-MSC treated Gp2: IP-MSC treated Gp3: Control group, diabetic (STZ),	4 weeks	-Effective attenuation of hyperglycemia in diabetic rats -Increased the plasma levels of rat insulin and c-peptide -Differentiated into IPCs in the diabetic pancreas -Promoted islet morphology, survival, and angiogenesis.
80	Male SD rats were intraperitoneally injected with 30 mg/kg of STZ for 3 consecutive days.	WJ-MSCs	A single dose of 5 × 10^6^ MSCs/rat	Tail vein	Gp1: WJ-MSC-derived-IPC-treated Gp2: Un Differentiated WJ-MSC-treated Gp3: STZ + NS	8 weeks Post-MSC transplant	-WJ-MSC-derived-IPCs via the continuous secretion of insulin improved BGL. -Undifferentiated hWJ-MSCs significantly improved insulitis and re-balance the inflammatory condition, with only a slight improvement in BGLs
81	Male SD rats were Injected intraperitoneally with a single dose of 60 mg/kg STZ	WJ-MSCs	Two doses 2 × 10⁶ MSCs/kg (With a three-weeks interval) MSCs were injected Three weeks after STZ injection	IP	Gp1: MSC-treated Gp2: Insulin & MSC-treated Gp3: Insulin, metformin & MSC-treated Gp4: insulin-treated Gp5: insulin & metformin-treated Gp6: diabetes controls Gp7: Healthy controls	Almost 6 weeks	-WJ-MSCs, either alone or combined with insulin, improved the symptoms of experimental diabetes via: • enhancing leptin signaling in the hypothalamus that affected the NPY/AgRP axis and the melanocortin-dependent mechanism in the brain. • Changing leptin signaling to increase energy expenditure and weight gain in T1D rats.
82	Twelve-week-old- NMRI nu/nu athymic mice were injected intraperitonially with 40 mg/kg of STZ for 3 days. (*T1D model*)	ASCs	A single dose of 0.5 × 10^6^ cells	IP or IV	Gp1: IP- MSC treated group Gp2: IV-MSC treated group Gp3: STZ group Gp4: Control group: No STZ, No MSCs	Almost 4 weeks post-transplant	-IP-MSC administrated achieved: -Glycemic control -Improved animals’ BW -Increased number of pancreatic islets proliferating cells via induction of the DLK1-ERK-FoxO1 signaling cascade in pancreas -Restored the host immune system balance (Th1/Th2 response)
83	Male NOD mice administered by the purified PD-L1 mAb [1000 µg on day 0, followed by 500 µg on days 2,5,7,9and12]. (*Cancer therapy -induced T1D model*)	ASCs	Repeated doses (1 × 10^6^ cells) at 2–3 days-interval for 2 weeks ASC administration was at the same days of PD-L1 mAb administration	Tail vein	Gp1: MSC (+) group: administered anti-PD-L1antibody plus MSC group Gp2: MSC (−) group: administered anti-PD-L1 antibody only Gp3: Control: no diabetes and no MSC	Almost 2 weeks after initiating anti-PD-L1 mAb treatments.	-MSCs prevented the PD-L1 mAb treatment-induced diabetes by 19% and significantly improved BGL in diabetic mice. -MSCs prevented the accumulation of CD3 T lymphocytes and CXCL9-positive macrophages that infiltrated into the fine gaps between the beta cells. -MSC administration increased the levels of murine plasma exosomes and altered the plasma cytokine profile.
84	Male SD rats Injected intravenously with a single dose of 50 mg/kg STZ (T1D model)	UC-MSCs	Multiple doses (once weekly for 4 weeks) ***Two regimens***: low dose (5 × 10^6^ cells /rat) vs. high dose (1 × 10^7^ cells/rat)	Tail vein	Gp1: low-dose UC-MSCs-treated Gp2: high-dose UC-MSCs-treated Gp3: STZ Gp4: Healthy	4 weeks treatment	-Reversed alterations in BW. -Reduced FBGL. -Decreased the serum ICAM-1 and VCAM-1 levels. -Improvement in aortic histopathology via ameliorating abnormal activation/phosphorylation of MAPK/ERK signaling in diabetic endothelium.
85	Female NOD/Ltj mice at ten–twelve weeks of age intraperitoneally injected with 40 mg/kg STZ for 5 days	UC-MSCs vs. BMSCs	A single dose of 1 × 10^6^ MSCs/mouse	Tail vein	Gp1: BM-MSC-treated Gp2: UC-MSC-treated Gp3: control group	2 weeks Post- MSC transplant	UC-MSC and BMSC comparably: -Significantly Decreased BGL to normal levels -Preserved beta-cell function -Reduced insulitis in diabetic pancreas -Upregulated the frequency of Tregs and reduced that of Th17 in spleen and PLN
45	Eight-week-old male C57BL/6J mice were injected intraperitoneally with a single dose of 100 mg/kg STZ	UC-MSCs	(Two doses) 1 × 10^6^ MSCs/mouse once weekly for 2 weeks	Tail vein	Gp1: UC-MSC-treated group Gp2: STZ + PBS Gp3: Healthy/normal mice	1 week Post-2nd dose MSC transplant	-MSCs migrated to liver, lung, pancreas, and kidney in STZ mice. -MSCs preserved islet morphologies, PDX-1 and GLUT2 expression levels, and GSIS, and thus improved islet function. -In vitro studies elucidated that UC-MSCs inhibited beta-cell apoptosis and oxidative stress via the Nrf2/HO-1 signaling pathway

*AF-MSCs* Amniotic fluid-derived derived mesenchymal stem/stromal cells (MSCs), *ASCs* Adipose tissue- derived MSCs, *AgRP* agouti-related protein, *BMSCs* Bone marrow-derived MSCs, *BGL* Blood glucose level, *BW* Body weight, *CXCL9* Chemokine ligand 9, *CD* Cluster of differentiation, *DCs* Dendritic cells, *DLK1* Delta like non-canonical Notch ligand 1, *DPSCs* Dental pulp stem cells, *ERK* Extracellular signal-regulated kinase, *FBGL* Fasting blood glucose level, *FOXO1* Forkhead box protein O 1, *FOXP3* Forkhead box P3, *GLUT2* Glucose transporter 2, *GSIS* Glucose-stimulated insulin secretion, *HO-1* Heme oxygenase 1, *ICAM-1* Intracellular adhesion molecule 1, *IR* Insulin resistance, *IV* Intravenous, *IP* Intrapancreatic, *IPCs* Insulin-producing cells, *IL-10* Interleukin 10, IL-6, Interleukin 6, *IL-1β* Interleukin 1 beta, *IL-2* Interleukin 2, *IL-17* Interleukin 17, *IL-4* Interleukin 4, *IFN-γ* Interferon gamma, *ILCs* Islet-like clusters, *IPGTT* Intraperitoneal glucose tolerance tests, *IPITTs* intraperitoneal insulin tolerance tests, *MAPK* Mitogen-activated protein kinase, *mAb* Monoclonal antibody, *NOD* Non-obese diabetic, *Nrf2* Nuclear factor erythroid 2-related factor, *NPY* neuropeptide Y, *NS* Normal saline, *OGTTs* oral glucose tolerance tests, *PL-MSCs* Placenta-derived MSCs, *PDX1* Pancreatic and duodenal homeobox 1, *PBS* Phosphate-buffered saline, *PD-L1* Programmed death ligand 1, *PI3K* Phosphatidyl inositol tri kinase, *PLN* Peripheral lymph nodes, *RBGL* Random blood glucose level, *SD* Sprague-Dawley, *STZ* Streptozotocin, *T1D* Type 1 diabetes mellitus, *Tregs* regulatory T cells, *Th1* T helper 1, *Th17* T helper 17, *TIMP-1* tissue inhibitor of metalloproteinase 1, *TNF-α* Tumor necrosis factor alpha, *TGF-β1* Transforming growth factor beta 1, *UC-MSCs* umbilical cord-derived MSCs, *VCAM-1* Vascular cell adhesion molecule 1, *VEGFA* Vascular endothelial growth factor A, *WJ-MSCs* Wharton’s Jelly-derived MSCs

In the context of hBMSCs, it has been reported that transplantation of hBMSCs elevated plasma and islet insulin contents in non-obese diabetic (NOD) mice with severe diabetes [32]. Relative to severe diabetic controls, hBMSC infusion decreased insulitis and reduced pancreatic TNF-α, while increased pancreatic TGF-β1 and IL-10 expression in NOD mice. Importantly, the MSC infusion increased the splenic Tregs percentages and levels of the plasma anti-inflammatory mediators; IL-4, IL-10 and TGF-β1, but reduced the percentages of splenic CD8^+^ T and levels of the plasma pro-inflammatory mediators; interferon gamma (IFN-γ), TNF-α and IL-17 A. Noteworthy, the reparative effects of hBMSCs tended to be dose-dependent, infusing multiple doses of hBMSCs had a longer therapeutic persistence, compared with a single dose regimen [32]. In another report, hBMSCs increased the frequency of the M2 macrophages and reduced that of the CD40-positive glucagon-producing α- cells in the islets of STZ- diabetic rats [79].

In the context of hASCs, their systemic administration enhanced the glucose tolerance, retained the beta-cell mass, and boosted the beta-cell proliferation in STZ-treated mice in a TIMP-1 dependent-effect [58]. TIMP-1 is a member of the matrix metalloproteinase (MMP) inhibitor family and its anti-apoptotic and regenerative effects in beta-cells have been identified [87]. Recently, Kawada-Horitani et al. [83]. found that systemic ASC treatment could prevent the development of immune checkpoint inhibitors-induced T1D in a NOD mouse model. Programed death 1 (PD-1)/PD-ligand 1 (PD-L1) blockade in cancer patients and NOD models developed T1D and hMSCs prevented the accumulation of CXCL9-positive macrophages infiltrated into the intricate gaps between the beta-cells. Additionally, MSCs significantly attenuated the infiltration of T cells into pancreatic beta-cells. Mechanistically, ASCs strongly increased plasma exosome levels and changed plasma cytokine profiles. Such findings suggest that ASC transplantation may be administered an adjuvant with cancer immune checkpoint cancer therapy [83]

Another MSC type is is human DP-derived stem cells (hDPSCs), which successfully improved hyperglycemia and induced beta-cell repair in STZ- diabetic rats through their ability to differentiate into IPCs. In addition, they seemed to inhibit beta-cell apoptosis, and to promote angiogenesis, as represented by by downregulation of caspase 3 (CASP3) and upregulated expression of VEGF, respectively [57]. Another limitedly investigated source is the AF, Villani and her colleagues [74] found that intracardiac injection of AF-derived MSCs (AF-MSCs) preserved and promoted endogenous beta-cell functionality and proliferation in STZ-NOD mice. Importantly, he protective role of AF-MSC was evident when stem cell transplantation is performed before severe hyperglycemia occurs, and BGL at the time of injection correlated with the pre-clinical response to AF-MSC injection, which suggests the importance of early MSC intervention for the best outcomes.

The most prevailing MSC type in experimental T1D therapy is UC-MSCs or Wharton’s jelly (WJ)-MSCs (around 50% of the included preclinical hMSC-T1D studies) [41, 45, 72, 73, 75, 78, 80, 81, 84, 85]. Administration of WJ-MSCs alone or in combination with insulin ameliorated the signs of experimental T1D by enhancing the leptin signaling in the hypothalamus and consequently affecting the neuropeptide Y (NPY)/AgRP axis and the melanocortin-dependent mechanism in the brain [81]. In another report, MSCs from BM and UC showed comparable abilities to regulate BGL and preserve beta-cell functions in T1D model. However, the beta-cell mass appeared higher in UC-MSC treatment than BMSCs, without statistical significance [85]. Such effect may be due to the stronger immunosuppressive ability of UC-MSCs than BMSCs [88]. In accordance, diverse authors ascribed the therapeutic potential of WJ-MSCs to their potent immunomodulatory functions in suppressing the inflammatory T-cell-dependent destruction of beta cells and promoting the tolerance in T1D models [41, 73, 75, 78, 80].

Among the variables which determine the outcome of MSC in T1D therapy is the route of administration. MSC injection in the tail vein of T1D models predominated and few studies compared the efficacy of different administration routes [72, 79, 82]. STZ- diabetic rats were injected with UC-MSCs (5 × 10^6^) via intravenous (IV/systemically) or intrapancreatic (IP/locally) routes [72]. The IP injection had less efficacy than the IV MSC transfusion. IV injected MSCs might migrate via the bloodstream to any injured tissues, consequently, they promoted pancreas regeneration, decreased and stabilized BGL, and improved the survival of diabetic mice. However, the IP MSCs exhibited local limited potential and reduced existence due to the harmful impact of pancreatic protease [72]. In another report, A contradictory data were reported when hBMSCs (1 × 10^6^ cells) were injected IP and IV in STZ-induced diabetic mice on day 7 of disease induction [79]. Local delivery, not IV, significantly reduced BGL on day 28 post-STZ injection. Interestingly, MSCs that were injected twice into the pancreatic region on days 7 and 28 reduced the BGL to borderline diabetic levels on day 56 as well as increased the body weight. Additionally, hBMSC-IP injected animals had an improved plasma insulin level, pancreas weight, and histomorphological level of islets including the number, size, and insulin immunoreactions compared with vehicle-injected mice [79]. In cope, hASCs administered IP, not IV, in STZ-diabetes dramatically increased the amount of replicating islet cells, islet area and number, the level of epidermal growth factor (EGF) gene, and Th1/Th2 response balance, which in turn improved both glycemic control and the animals’ body weight. The reparative effects of IP injected hASCs was mediated via inducing the pancreatic DLK1/EGF-ERK-FoxO1 signaling cascade which promoted the anti-apoptosis BCL-2/BAX ratio in STZ- murine pancreas. In vitro studies revealed that the physical contact between hASCs and murine pancreatic beta-cells is essential for ASC protective effect through the AKT and ERK pathway [82].

Other determinant variables in MSC-based therapy in experimental T1D are the dose amount (low vs. high) [32, 77], and the dose frequency (single vs. repeated administration). It has been illustrated that the high dose of hASCs (2 × 10^6^) induced a stronger anti-hyperglycemic and survival effects in STZ-T1D model than the respective lower dose (1 × 10^6^) [77]. Despite most of the research applied the single dose regimen, those studies injected multiple bolus of MSCs in experimental T1D confirmed prolonged/sustained anti-diabetic effects in the multiple-doses-treated groups, compared with the single-regimen groups, in which the reparative effects were transient [32, 55]. Noteworthy, only two studies compared the differential effect of undifferentiated MSCs and their IPC-derivatives using the same experimental T1D setup [51, 80]. Kadam et al. [51]. reported the comparable potential of placenta-derived MSCs (PL-MSCs) and their Islet-like clusters in ameliorating hyperglycemia in STZ-diabetic mice, suggesting the in vivo differentiation of PL-MSCs into IPCs. In another report, differential therapeutic mechanisms of IV transplanted hWJ-MSCs and their IPCs were detected in a STZ-diabetic rat [80]. The WJ-MSC-IPCs survived in the pancreatic islets of the rats and significantly reduced BGL and improved glucose metabolism by the continuous secretion of insulin. However, the undifferentiated hWJ-MSCs improved the ability of injured islets to secrete insulin by restoring immune balance in the diabetic rats, with less pronounced improvements in the BGL. The anti-inflammatory effect of WJ-MSCs in vivo was evidenced by reducing the serum level of IFN-γ and IL-1β and increasing the level of IL-4 and TGF-β [80].

Interestingly, not only MSCs, derived from healthy donors, but also those derived from patients with newly diagnosed T1D were effective in preventing the death of pancreatic beta-cells and promoting the reversal of hyperglycemia in STZ-diabetic rat. BMSCs derived from healthy donors or patients with newly diagnosed T1D significantly reduced pancreatic inflammation, preserving existing and newly formed beta-cells, leading to regular insulin production [76]. This anti-inflammatory effect was evident as levels of IL-2 and IFN-γ were decreased in the pancreatic tissue of mice treated with both MSC types on 35 days post-therapy. Furthermore, there was a slight, though not significant, decrease in levels of IL-6, TNFα, and IL-17 in the pancreatic tissue following MSC injection [76]. Importantly, hMSCs delayed the onset of autoimmune diabetes in NOD mice via inhibiting Th1 polarization, however, promoting Treg generation, in TSG-6 dependent mechanism and the results might indicate the preventive function which MSC infusion or recombinant human TSG-6 could play in susceptible T1D individuals [38].

### Preclinical evidence for the anti-T2D potential of undifferentiated hMSCs

The preclinical efficacy of hMSCs, derived from different sources, in T2D has been evidenced by many research groups [34–36, 39, 40, 43, 47–49, 56, 62, 63, 89–95] (Table 2). T2D has been induced by a high-fat diet (HFD) (40% fat, 41% carbohydrate, and 19% protein), followed by injecting a single dose of STZ with a broad range (25-100 mg/kg body weight). Genetically modified (db/db) mice with leptin receptor deficiency and then spontaneously develop hyperphagia-induced hyperglycemia, IR, and dyslipidemia have also been used as a model of obesity-induced T2D [86]. Another model known as WNIN/GR-Ob rat model (WNIN mutant Obese rats) has been introduced [40]. It closely resembles pre-clinical /clinical obese diabetic subjects presenting metabolic dysfunctions like impaired glucose disposal, IR, increase body mass index (BMI), osteoarthritis, hypertriglyceridemia, as well as hypercholesterolemia [40].

**Table 2:** Undifferentiated hMSC-based therapy in experimental T2D

Ref.	DM model Induction	MSC source	Cell dose & Frequency	Route of administration	Study Experimental Groups	End of Follow up	Main findings
89	Seven-week-old male Sprague-Dawley rats were fed normal chow or HFD for 2 weeks and then injected with STZ, 50 mg/kg, IV). (*T2D model*)	BMSCs	A dose of (2 × 10^6^ cells/rat) at 7 (*early phase*) or 21 days (*late phase*) of STZ injection. The early phase rats, were then secondly infused at 42 days post-STZ	Tail vein	Gp1: MSC-treated T2D rats’ group Gp2: untreated T2D rats’ group Gp3: Normal group	6 weeks post MSC-2nd injection	-Ameliorated hyperglycemia -Improved GLUT4-mediated insulin sensitivity - better outcome in the early phase injection and with double, instead of single dose injection.
56	Seven-week-old male B6 mice which were continuously fed HFD for 6 months (*T2D model*)	ASCs	Two doses (4.2 × 10^7^ cells/kg/each time) with 2 months-interval)	Tail vein	Gp1: Mice-fed ND and PBS GP2: Mice-fed ND and MSCs Gp3: mice-fed HFD and PBS GP4: mice-fed HFD and MSCs	4 weeks Post MSC 2nd transplant	-Restoring glucose homeostasis -Increase insulin levels via differentiation into IPCs in vivo -Enhancing glucose tolerance and improving insulin sensitivity via IL-1RA-dependent mechanism. -Reducing systemic Inflammation -Prevention of fatty liver development and restoration of hepatic glycogen storage.
49	Eight-week-old male Sprague-Dawley rats were fed HFD for 5 weeks, and then injected with STZ, 40 mg/kg, intraperitoneally (*T2D model*)	BMSCs	A single dose of 2 × 10^6^ MSCs injected 7 days after STZ administration	Tail vein	Gp1: MSC-treated T2D Gp2: untreated T2D Gp3: Healthy group	NA	-Ameliorated hyperglycemia -Promoted restoration of pancreatic beta-cells -Enhanced formation of autolysosomes -Augmented autophagy -Reduced beta-cell apoptosis and reduced ROS generation -Consequently, Increased number of insulin granules in pancreas.
34	Eight-week-old male Sprague-Dawley (SD) rats were fed HFD or a ND, followed by injection of 25 mg/kg STZ (*T2D model)*	UC-MSCs	A single dose of 3 × 10^6^/rat (Day 7 post STZ injection)	Tail vein	Gp1: MSC- Treated Gp2: Control	NA	-Improved glucose homeostasis and IR via production of IL-6 by UC-MSCs Which induced ATMs toward M2 phenotype.
90	Male Sprague–Dawley (SD) rats were fed HFD diet for 8 weeks, followed by intraperitoneal injection of STZ (25 mg/kg (*T2D model)*	UC-MSCs	A single dose of 3 × 10^6^ MSCs/rat (7 days post STZ injection)	Tail vein	Gp1: UC-MSC-treated Gp2: T2D + PBS (diabetic control)	NA	-Ameliorated hyperglycemia - Inhibited the NLRP3 inflammasome activation and improved insulin sensitivity
91	Eight-week-old male C57BL/6J mice were fed HFD for 12 weeks, followed by a single intraperitoneal dose of 100 mg/kg STZ	UC-MSCs	A single dose of 1 × 10^6^ hUC-MSCs/mouse	Tail vein	Gp1: UC-MSC-treated Gp2: diabetic + PBS	Almost 4 weeks Post-MSC transplant	- Induced M2 macrophage polarization in pancreatic islets via IL-6 and MCP-1- dependent mechanism
92	Eight-week-old male C57BL/6 mice were given HFD for 8 weeks, followed by a single intraperitoneal dose of 100 mg/kg STZ	UC-MSCs	A single dose of 1 × 10^6^ hUC-MSCs/mouse	Tail vein	Gp1: MSC group Gp2: DAC group Gp3: MSC + DAC group Gp4: T2D group + PBBS	4 weeks post MSC-transplant	-DAC sustained the anti-diabetic (anti-hyperglycemic and anti-IR) effects of MSCs. -The combined therapy induced greater induction of ATMs into M2 via IL-4R/STAT6/PPAR-γ dependent mechanism, compared with the individual regimens
47	GK rats (twelve-weeks old, male) consumed a HFD for 4 weeks (*T2D model*)	SHED Vs BMSCs	A Single dose of 4 × 10^6^ cells	Tail vein	Gp1: SHED-treated Gp2: BMSC-treated Gp3: diabetic rats + PBS (4) Healthy controls	8 weeks post MSC- transplant	-Improved Glucose Homeostasis -Restored pancreatic islet and liver -Reversal of the diabetic-induced increase in G-6-Pase and Pck1, however, the diabetic-induced decrease of GSK3b, GLUT2, PFKL, PFKM, and PK at gene and/or protein levels. -Those changes were associated with: • Increased glycogen synthesis and activated glycolysis in the liver of GK rats. • Decreased gluconeogenesis (production of glucose from non-carbohydrate sources) in the liver. • Decreased liver IR
43	Eight-week-old male SD rat which were fed HFD for 8 weeks, followed by a single intraperitoneal dose of STZ (25 mg/kg). (*T2D model*)	ASCs	Multiple doses of (3 × 10^6^ cells) [once weekly for 24weeks]	Tail vein	Gp1:MSC-treated group Gp2:T2DM group (PBS only)	On week 57 (after 24 times of MSC treatment)	Restoring Glucose homeostasis - (Improved HOMA-IR. -Enhanced beta-cell Function (improved HOMA-β) -Ameliorating T2D complication in kidney, liver, or lung -Potentiated anti-inflammatory effects
59	Male Sprague-Dawley (SD) rats (6 weeks old, weight 180–220 g) were fed high fat and high glucose diet for 8 weeks, followed by a single intraperitoneal dose of STZ (30 mg/kg). (*T2D model*)	UC-MSCs Alone or in combinate-ion with liraglutide	Two doses (1 × 10^6^ cells/mouse) With one-month interval	Tail vein	Gp1: MSC group Gp2: Liraglutide group Gp3: MSC + Liraglutide Gp4: T2D group	8 weeks	-The combined therapy exhibited superior ability to reduce BGL, BW, improve islet morphology, increase islet insulin content and significantly decrease beta-cell apoptosis in a ASK1/JNK/BAX pathway-dependent manner
94	Six-month-old WNIN/Control and WNIN/GR-Ob Rats (*Ob-T2D model*)	PL-MSCs	Three doses (once weekly) Of 1 × 10^6^ MSCs/rat	IM	Gp1: WNIN/GR-Ob (Ob-T2D) + PL-MSC-treated Gp2: WNIN/Control + PL-MSCs GP3: WNIN/GR-Ob (Ob-T2D) + PBS GP4: WNIN/GR-Ob (Ob-T2D) + pbs	7 weeks post MSC-3rd transplant	-Restored HOMA-IR and glucose homeostasis -Regulated glucose utilization via activating PI3K/AKT pathway in AT of treated rats.
62	Two models. Eight-week-old male C57BL/6J mice were given HFD for 12 weeks, followed by a single intraperitoneal dose of 100 mg/kg STZ (1) & genetically obese, leptin receptor-deficient db/db mice, a spontaneous T2DM model (2)	UC-MSCs	**In model 1** Two doses of 1 × 10^6^ MSCs/mouse (Once weekly) **In model 2** six doses of 1 × 10^6^ MSCs/mouse (Once weekly) *Therapy was injected at the peak of beta-cell dedifferentiation in model 1* *and at the early (at the age of 12 weeks) and late (at the age of 30 weeks) stages of beta-cell dedifferentiation in model 2*	Tail vein	Gp1: model 1 (UC-MSC-treated) Gp2: model 2 (UC-MSC-treated) Gp3: diabetic control model 1 + PBS Gp4: diabetic control model 2 + PBS Gp5: Healthy normal mice (C57BL/6J mice)	1-week After the last dose of MSC transplant	-Reversed beta-cell dedifferentiation -UC-MSCs significantly improved glucose and lipid homeostasis in db/db mice in the early treatment group, with a significant reversal of beta-cell differentiation. -Partial reversal of beta-cell dedifferentiation in the late treatment group.
39	C57BL/6 db/db mice (*T2D model*)	DPSCs, WJ-MSCs, or ASCs	Three doses once every 2-weeks ***Three regimens*** 0.25 × 10^6^ (Low dose) 0.50 × 10^6^ (Medium dose), or 1 × 10^6^ (High dose)	Tail vein	Gp1: db/db mice + UC-MSCs Gp2: db/db + DPSCs Gp3: db/db + ASCs, Gp4: db/db + Metformin Gp5: db/db + vehicle Gp6: wild type + vehicle	6 weeks post MSC- transplant	-UC-MSCs showed the super efficacy in reducing FBGL, increasing fasting insulin levels, and improving glucose and insulin tolerance in a dose-dependent manner, whereas DPSCs showed an intermediate efficacy and ASCs showed the least efficacy on these parameters. - UC-MSCs reduced the serum LDL-C levels with the most prominent potency, however, hASCs had only very weak effect on LDL-C. - ASCs substantially reduced the lipid content and histological lesion of liver and accompanying biomarkers of liver injury such as serum AST and ALT levels, whereas UC-MSCs and DPSCs displayed no or modest effects on these parameters, respectively.
95	**Two models** Four-week-old male C57/BL6 mice were fed HFD for 16 weeks to induce DIO & 5- week-old male diabetic db/db (BKS.Cg-Dock7m +/+ Leprdb/J) mice (T2D model).	UC-MSCs	Three doses of 1 × 10^6^ MSCs/mouse On days 1, 30, and 60 of the beginning of FMD	Tail vein	Gp1: UC-MSC-treated Gp2: FMD group Gp3: UC-MSC + FMD Gp4: diabetic or DIO mice controls	11 weeks	-An additive effect of UC-MSCs to FMD cycles in ameliorating hepatic steatosis and dyslipidemia, however, no additive effect was observed regarding the anti-hyperglycemic UC-MSCs ability in the presence of FMD cycles.
40	Six-month-old female WNIN/GR-Ob (Ob-T2D) rats Vs WNIN/Control rats	PL-MSCs	Three doses of 1 × 10^6^ / rate (Once weekly)	IM	Gp1: WNIN/GR-Ob + MSCs Gp2: WNIN/control + MSCs Gp3: diabetic control + PBS Gp4: Normal control + PBS	7 weeks Post-3rd MSC transplant	- Peripheral blood glucose clearance -Restored HOMA-IR, re-establish dysregulated cytokines and PI3K-Akt pathway, in skeletal muscle -Enhanced Glut4 expression and glucose uptake.
48	Male seven-week-old BKS-Lepr^em2Cd479^/Nju ^(db/db)^ mice (T2D ^db/db^ mice) C57BLKS/JNju (normal)	AM-MSCs Vs UC-MSCs (The same donor)	A single dose of 1.5 × 10^6^ cells/mouse	IV	Gp1: AM-MSC-treated Gp2: UC-MSC-treated Gp3: T2D mice + NS Gp4: Normal mice + NS	5 weeks Post-MSC transplant	-AM-MSCs or UC- MSCs could comparably -Reduce hyperglycemia and improve IR - AM-MSC or UC-MSC infusion could improve glycolipid metabolism in the liver of db/db mice, which was evidenced by: • Decreased liver to BW ratio, • Reduced lipid accumulation, • Upregulated glycogen synthesis, - Increased Akt phosphorylation.
35	Eight-week-old male C57BL/6 mice were given HFD for 8 weeks, followed by a single intraperitoneal dose of 85 mg/kg STZ	UC-MSCs	Six doses of of 1 × 10^6^ cells/mouse (Once weekly)	Tail vein	Gp1: UC-MSC-treated Gp2: diabetic control	1 week after the last MSCs injection	-ATMs polarization into M2 phenotype mixed with four sub-phenotypes. -Investigating AT M2 subpopulations via SMART RNA-sequencing and heatmap clustering revealed that M2a and M2c subphenotypes predominated, while M2b and M2d (tumor-associated macrophages) exhibited a decreasing trend after infusion of MSCs. - Lower expression levels of tumor- genes associated with tumor, inflammation, or fibrosis, in MSC-treated group in comparison to the T2D control group.
99	Six-week-old male C57BL/6 mice fed HFD for continuous 10 weeks (DIO model)	ASCs	5 × 10^5^ cells/ mouse	IM	Gp1: ASC-treated Gp2: MetASC-treated Gp3: metformin-treated Gp4: lean control Gp5: DIO control	4 weeks post MSC- transplant	-Reduced systemic inflammation (serum IL-6 level) and expression of inflammation genes (*IL-6* and *PAI-1*) in the liver. Upregulated glucose uptake and GLUT4 expression in skeletal muscles -Reversal of hyperglycemia, hyperinsulinemia, and triglyceremia
102	Four-week-old male C57BL/6J mice fed HFD for 30 weeks (DIO model)	ASCs	A total of 5 × 10^6^ cells administered over the course of 10 weeks	IV	Gp1: MSC-treated Gp2: M-BA-treated Gp3: MSC-Lysate-treated Gp4: diabetic control	10 weeks	- Suppression of HFD-induced obesity and HFD-associated lipid metabolic syndrome. Attenuated HFD-induced liver fibrosis and inflammation. -Repressed glucose intolerance - Altering the expression pattern of PPARs and regulating the adipokines expression
101	C57BL/6J male mice (seven-week-old) were fed HFD for 16 weeks to provoke (DIO model)	ASCs	Three doses of 2 × 10^6^ ASCs/mouse (Once weekly)	Tail vein	Gp1: IB-hASC infusion, Gp2: W-hASC (from vWAT) infusion Gp3: diabetic control, NS infusion	3 weeks post MSC- transplant	-Reduced weight gain and improved glucose tolerance. - Modulated lipid metabolism and induced browning of AT, as indicated by upregulated expression of UCP1. - Reduced inflammatory gene expression in AT of treated mice. -Infusion of IB-hASCs was superior to W-hASCs in suppressing lipogenic and inflammatory markers, as well as preserving insulin secretion.

*ASCs* Adipose tissue-derived mesenchymal stem/stromal cells (MSCs), *AM-MSCs* Amniotic membrane-derived MSCs, *ATMs* Adipose-tissue macrophages, *ALT* alanine aminotransferase, *AST* aspartate transaminase, *ASK1* Apoptosis signal-regulating kinase 1, *BAX* BCL2 associated X, apoptosis regulator, *BMSCs* Bone marrow-derived MSCs, *BW* Body weight *DAC* Decitabine, *DIO* Diet-induced obesity, *DPSCs* Dental pulp stem cells, *FBGL* Fasting blood glucose level, *FMD* Fasting-mimicking diet, *GK rat* Goto Kakizaki rat, *GLUT2* glucose transporter 2, *GLUT4* Glucose transporter 4, *G-6-Pase* glucose-6-phosphatase, *GSK3β* glycogen synthase kinase 3 beta, *HFD* High-fat diet, *HFHF* high-fat high-fructose, *HOMA-IR* Homeostatic model assessment index of insulin resistance, *HOMA-β* Homeostatic model assessment index of beta-cells, *IR* Insulin resistance, *IV* Intravenous, *IM* intramuscular, IL-6, Interleukin 6, *IL-1β* Interleukin 1 beta, *IL-1RA* Interleukin 1 receptor antagonist, *IL-10* Interleukin 10, *IL-4R* Interleukin 4 receptor, *IPCs* insulin-producing cells *IPGTT* Intraperitoneal glucose tolerance tests, *IPITTs* intraperitoneal insulin tolerance tests, *IB-hASCs* hASCs isolated from the adipose tissue surrounding a pheochromocytoma, *JNK* c-Jun N-terminal kinases, *LDL-C* low-density lipoprotein cholesterol, *M2* anti-inflammatory macrophages, M-BA, MSC-derived brown adipocytes, *MCP-1* Monocyte chemotactic protein 1, *metASCs* Metformin-treated SCs, *NA* Non-applicable, *ND* Normal diet, Ngn, Neurogenin, *NLRP3* nucleotide-binding domain, leucine-rich–containing family, pyrin domain–containing-3, *NS* Normal saline, *OGTTs* oral glucose tolerance tests, *Ob-T2D* T2D-induced by obesity, *PL-MSCs* placenta-derived MSCs, *PAI-1* Plasminogen activator inhibitor 1, *PBS* Phosphate-buffered saline, *Pck1* phosphoenolpyruvate carboxykinase 1, *PK* pyruvate kinase, *PFKL* phosphofructokinase liver type, *PFKM* 6-phosphofructokinase, *PI3K* Phosphoinositide 3-kinases, *PPAR-γ* peroxisome proliferator-activated receptor gamma, *RBGL* Random blood glucose level, *ROS* Reactive oxygen species, *SHED* Stem cells from human exfoliated deciduous teeth, *SD* Sprague-Dawley, *STZ* Streptozotocin, *STAT6* Signal transducer and activator of transcription 6, *T2D* Type 2 diabetes mellitus, *TNF-α* Tumor necrosis factor alpha, *UC-MSCs* umbilical cord-derived MSCs, *UCP1* Uncoupling protein 1, *WJ-MSCs* Wharton’s Jelly-derived MSCs, W-hASCs, ASCs isolated from visceral adipose tissue from lean and healthy subjects

Injecting hBMSCs in HFD/STZ-T2D model, during different disease phases: early (at day 7) vs. late (at day 21) of STZ injection, showed positive impact on beta-cell insulin content, during the early phase treatment. However, IR was improved during both therapy phases, leading to reversal of hyperglycemia [89]. Noteworthy, the decline of hyperglycemia in the studied T2D model was transient with a single bolus of MSCs (≤ 4 weeks), however, second injection induced a better reduction in hyperglycemia, than the first dose, which sustained over longer duration [89]. In another report, hBM-MSC infusion augmented autophagy in beta-cells of T2D model, as represented by increased expression of lysosome-associated membrane protein 2 (LAMP2) and enhanced formation of autophagosomes and autolysosomes. That was associated with significantly improved mitochondrial functions and increased insulin granules number [47]. As well, hMSCs isolated from human orbital fat tissues were able to correct the inflammatory and metabolic imbalances in HFD diabetic mice [56]. The hASCs supported pancreatic islet growth by direct differentiation into IPCs and by mitigating the cytotoxicity of IL-1 and TNF-α in the pancreas. Human IDO, IL-10 and soluble neutralizing TNF receptor (TNF RII) genes were upregulated in the treated mice pancreatic tissues. hASCs improved glucose tolerance and that was correlated with their localization in the liver and skeletal muscle. In the liver, ASCs improved insulin sensitivity by preventing fatty liver formation as well as restoring glycogen storage in hepatocytes. Intriguingly, systemic ASC transplantation did not alter adipocyte number, but it decreased inflammatory cell infiltration in AT of diabetic mice and reduced serum levels of adipokines, including leptin and TNF-α contributing to inhibition of inflammation in AT of obesity-induced diabetes. Leptin is an adipokine mainly secreted by white AT, and its circulating level is proportional to the total amount of fat in the body. It also acts as a proinflammatory and mitogenic factor for immune cells, it is thus a marker of AT–inflammation [96].

In a Goto-Kakizaki (GK) rat (non-obese T2D model), administration of MSCs from human exfoliated deciduous teeth (SHED) effectively reversed hyperglycemia and restored the function and architecture of pancreatic islets. MSC administration selectively acts on different key enzymes that play important roles in glycogen synthesis and gluconeogenesis for the favor of increased glycogen synthesis and decreased gluconeogenesis and IR in liver of GK rats [47]. Not only pancreas, liver, and AT are the target organs affected by MSC therapy, but also the skeletal muscles do. PL-MSC therapy remodeled the cytokine efflux and insulin signaling, in addition to enhanced Glut4 expression and glucose uptake, in the skeletal muscle of WNIN/GR-Ob- T2D rats [40]. Kotikalapudi et al. [40]. reported a significant decrease in the level of pro-inflammatory cytokines (IL-1β, IL-6, TNF-α, MCP-1, IFN-γ, IL-18) and significant increase of anti-inflammatory cytokines (IL-10, IL-4, IL-13, GM-CSF, TGF-β), in addition to VEGF and leptin in the skeletal muscle after PL-MSC local injection. Leptin increases fatty acid oxidation and decreases esterification, reducing IR in skeletal muscle [97]. As well, hASCs upregulated glucose uptake in experimentally T2D skeletal muscles by IL-1RA- associated GLUT4 increased expression [56]. IL-1-RA has been found to be a diabetogenic modulator produced by MSCs [98]. At systemic level, ASCs increased the anti-inflammatory cytokine IL-10 and inhibited the expression of IL-6, IL-1β, and TNF-α [43].

Comparative studies for the efficacy of MSCs from different human tissues in the same T2D experimental setup have been limitedly conducted [39, 48]. The differential effectiveness of MSCs derived from DP, AT, or UC in treating glucose and lipid metabolic problems in db/db mice has been tested [39]. IV injection of hUC-MSCs, DPSCs, and ASCs into T2D mice demonstrated that the three kinds of MSCs may be useful treatments for T2D and its associated lipid dysregulation, and UC-MSCs are superiorly effective in improving hyperglycemia, glucose intolerance, IR, and dyslipidemia. Whereas, ASCs are more effective in reducing liver fat content and hepatic injury. In that study, dose-dependent amelioration of hyperglycemia was reported, where high (1 × 10^6^) induced better effects than intermediate (0.5 × 10^6^) and low (0.25 × 10^6^) MSC doses [39]. Another report illustrated a comparable anti-diabetic potential of UC-MSCs and amniotic membrane (AM)-derived MSCs in T2D db/db mice and that was ascribed to the improved glycolipid metabolism, increased insulin sensitivity, and decreased inflammation in the liver of db/db mice [48]. AM-MSCs and UC-MSCs have been suggested as a very promising therapeutic agents to treat metabolic dysfunction-associated fatty liver diseases, and that may be attributed to IL-6 secretion by MSCs, however, further research is needed to verify this hypothesis [48].

Numerous reports demonstrated that hMSCs ameliorate IR in T2D models via potentiating polarization of adipose tissue macrophages (ATMs) [34, 35, 92], or intra-islet macrophages [36, 91], toward M2 anti-inflammatory phenotype. hUC-MSC induced M2 macrophages differentiation via partially increased IL-6 production, which in turn enhanced IL-4R expression in macrophages making them more sensitive to IL-4/IL-13 signaling, and so to M2 polarization [34]. In addition to IL-6, hUC-MSC infusion induced M2 macrophage differentiation in islets of T2D mice via MCP-1 dependent mechanism [91]. The additive potential of acombined therapy of UC-MSCs and a low dose of decitabine (0.25 mg/kg DAC for 5 consecutive days) in T2D models has been proved [36, 92]. Decitabine, an FDA-approved DNA methyltransferase (DNMT) inhibitor, an epigenetic modifier which is often used in the treatment of hematological disease [36]. The combined therapy induced greater ATMs [92], or intraislet macrophages [36], polarization into M2, compared with the individual regimens, via the IL-4R/STAT6 axis in a peroxisome proliferator-activated receptor gamma (PPAR-γ)-dependent manner or activated PI3K/AKT pathways, respectively, in macrophages. Recent insights demonstrate that the systemic administration of UC-MSCs in T2D model directed ATMs into the M2 phenotype mixed with four sub-phenotypes [35]. Investigating AT M2 subpopulations via SMART RNA-sequencing (RNA-seq) and heatmap clustering revealed that M2a and M2c subphenotypes predominated, while M2b and M2d (tumor-associated macrophages) exhibited a decreasing trend after infusion of MSCs. Importantly, the MSCs group, compared with the diabetic control group, did not appear to express higher levels of genes associated with tumor, inflammation, or fibrosis, in comparison to the T2D control group. Such deep analysis supports a hybridity state of four M2 sub-phenotypes, in AT of T2D model after MSC infusion [35]. As well, hUC-MSCs have been reported to improve insulin sensitivity in target tissues of T2D through inhibiting the NLRP3 inflammasome activation [90].

Several reports demonstrate that administration of hMSCs, specifically hASCs, in murine models of diet-induced obesity (DIO) can reduce obesity associated- altered glucose metabolism and IR [99–103]. ASC-mediated amelioration of skeletal muscle IR was attributed to upregulation of miRNA-206, which promotes muscle regeneration, expression of myoblast determination protein (MyoD) and increase the protein content of the skeletal muscle of a DIO-associated metabolic disturbance model [100]. Calvo et al. [101]. compared the anti-diabetic and anti-obesity effects of hASCs isolated the AT surrounding a pheochromocytoma, as an inducible brown fat, (IB-hASCs) relative to those isolated from visceral AT from lean and healthy subjects (W-hASCs) in a murine model of DIO. It has been found that both ASC therapies mitigated the metabolic abnormalities of obesity to a similar extent, including reducing weight gain and improving glucose tolerance. However, infusion of IB-hASCs was superior to W-hASCs in suppressing lipogenic and inflammatory markers, as well as preserving insulin secretion. These findings provide evidence for the metabolic benefits of visceral ASC infusion and support further studies on IB-hASCs as a therapeutic option for obesity-related metabolic dysregulations. Lee et al. [102]. demonstrated that MSC–based therapies can ameliorate obesity-related nonalcoholic fatty liver disease, nonalcoholic steatohepatitis, glucose intolerance, and inflammation. In that study, the effectiveness of hASCs, ASC-derived brown adipocytes (M-BA), and MSC lysate was compared after IV transplantation into obese mice. All 3 MSC-based treatments improved obesity-associated metabolic syndromes after repeated administration for 10 weeks. MSC-based treatments altered the ratio of adiponectin to leptin and regulated the expression of PPAR-α and PPAR-γ, which are involved in maintaining energy homeostasis, in major metabolic tissues. Among treatments, M-BA showed the strongest beneficial effect. Importantly, M-BA administration not only reduced obesity-associated metabolic syndromes but also reduced body weight and hyperlipidemia, indicating that it is an effective therapy for obesity. All the above presented preclinical data demonstrate the possible benefits of the application of none genetically engineered MSCs derived from different human tissues for the treatment of T1D, T2D, or obesity-induced metabolic syndromes and give new insight on the mechanism by which the beneficial effects are achieved.

### Preclinical anti-diabetic evidence of hMSC-derived extracellular vesicles

The hMSC-derived extracellular vesicles (hMSC-EVs), including exosomes (MSC-EX), microvesicles, and apoptotic bodies, contribute to the hMSC therapeutic functions [104]. Comprehensive reviews illustrating MSC-EVs biogenesis, contents, and characteristics are recommended [104, 105]. The application of hMSC-EVs as the main cell-free therapy for experimental T1D [106–109], or T2D [110–112], treatment is becoming more and more extensive [104, 109].

In the context of T1D, hMSC-EVs have been reported to induce pleiotropic immunoregulatory effects for the favor of tolerogenic systemic and pancreatic environment. Favaro and her associates reported that hMSC-EVs promoted the regulatory anti-inflammatory (IL-10 producing) phenotype of dendritic cells [DCs; antigen-presenting cells (APCs)] derived from patients with T1D [106]. In that study, MSC- and MSC-EV-conditioned DCs acquired an immature phenotype with reduced activation and increased IL-10 and IL-6 production. Conditioned DCs exhibited attenuated potential to prime T-cells toward an inflammatory phenotype. MSCs and their EVs can thus treat T1D by inducing the tolerance of DCs to inhibit aggressive T cell responses to islet antigens [106]. The immunomodulatory potentials of MSC-EVs to delay the onset of T1D in mice, via inhibiting the activation of APCs and suppressing the development of inflammatory Th1 and Th17 cells, have been confirmed [107]. Moreover, menstrual blood-derived MSC‐EX enhanced the beta‐cell mass and insulin production in the pancreas of STZ-diabetic animals that received repeated MSC-EX doses. Further investigations propose that exosomes induced the islet regeneration through pancreatic and duodenal homeobox 1(PDX-1)-dependent pathway [108]. PDX-1 is a master transcription factor orchestrates the beta‐cell differentiation and survival [113]. Interestingly, ASC- EX loaded with nano-selenium, exhibited marked pancreatic regenerative, antioxidant, immunomodulatory, anti-inflammatory, and anti-apoptotic capacities in STZ-induced T1D, compared to those loaded with elemental selenium, a natural antioxidant [109].

In the context of T2D, the therapeutic effect of hUC-MSC- small EVs and EX has been investigated [110, 111]. hUC-MSC-EX maintained glucose homeostasis via different mechanisms; (1) they restored the phosphorylation (tyrosine site) of IRS-1 and AKT in insulin target tissues, (2) they promoted expression and membrane translocation of GLUT4 in muscle and (3) they inhibited glycogenolysis in liver. Additionally, (4) hUC-MSC-EX abrogated STZ-induced beta-cell apoptosis to restore the insulin-secreting function. Apelin is an adipocyte-derived factor that shows promise in improving IR. Recently, it has been reported that WJ-MSC-derived EVs loaded with apelin showed enhanced capacity to improve insulin sensitivity in T2D mice, driven by a significant increase in the phosphorylated AKT and GLUT4 expression [112]. For safe and efficacious delivery strategies for MSC-EVs in diabetes therapies, it has been demonstrated that the minimally invasive I.V. approach would serve as a better delivery strategy, than intra-arterial route of administration, due to the higher spleen uptake, enhancing the immunomodulatory functions of the IV administered MSC-EVs [114].

The above studies provided promising results for the use of EX-cell free therapy in ameliorating T1D or T2D pathogenic mechanisms. Available data indicate that MSC-derived EX may be more safe, rapid and easier to inject with more efficient results than the MSCs themselves [113].

### Priming/preconditioning strategies that potentiate the anti-diabetic potential of hMSCs: bench and beside insights

The efficacy of MSC-based treatments in clinical trials greatly varies [115, 116], due to both intrinsic differences resulting from the choice of diverse cell sources and non-standardized production methods [117]. To minimize such limitation and to enhance MSC therapeutic potential, researchers have explored many priming/ preconditioning strategies, that can tailor MSC regenerative properties to specific medical conditions [118, 119]. Many hMSC priming manipulations have been introduced [118, 120], including, among others, exposure to inflammatory factors [24, 121–123], or small chemical molecules or biomolecules [33, 99, 124–130], genetic modification [131–141], or three-dimensional (3D) culture [142, 143]. We discuss some of the promising preconditioning approaches which can enhance the therapeutic efficacy of hMSCs in DM and they are summarized in Table 3.

**Table 3:** Priming strategies of hMSCs in the context of DM

Ref.	hMSC Type	Priming strategy & conditions	The potentiated biological characteristic or therapeutic Efficacy	Preclinical Evidence In T1D or T2D model
131	BMSCs	GE (overexpress VEGF, angiogenic factor)	Beta-cell regenerative capacity	√
132	HF-MSCs	GE (overexpress insulin with controlled release)	Anti-hyperglycemic effect	√
124	ASCs	SDF-1α TTT (Chemokine, 0.5 mg/l for 1–6 h)	Survival	√
99	ASCs	Metformin TTT (Insulin sensitizer, 1 mM for 16 h)	Anti-hyperglycemic, Anti-hyperlipidemic, Anti-hyperinsulinemia effect	√
133	ASCs	GE (overexpress betatrophin, hormone)	Beta-cell proliferation	√
134	WJ-MSCs	GE (overexpress apelin, insulin sensitizer)	Anti-IR potential in T2D	√
135	ASCs	GE (overexpress SOD-2 or Cat, antioxidant enzymes)	ASC potential to restore glucose tolerance and to suppress inflammation and liver fat accumulation in obese mice	√
140	ASCs	GE (overexpress sTNF-αR and HO-1, anti-oxidative stress mediators)	Porcine islet graft supportive ability	√
125	DPSCs	Resveratrol TTT (anti-oxidant, 50–100 μm for 1 h before TNF-α TTT)	Resistance to TNF-α induced inflammation at the concentration of 2.5 ng/mL	X
126	BMSCs	Resveratrol TTT (a potent SIRT1 activator that exerts anti-inflammation property, 50 μm for 24 h)	Mitigating TNF-α induced inflammation in MSCs	X
16	ASCs (From healthy or T2D patients)	IFN-γ (Inflammatory cytokine, 100ng/ml for 48 h)	Immunomodulatory properties	X
136	UC-MSCs	GE (overexpress TIMP-1, a regulator of cell proliferation & apoptosis)	Beta-cell regenerative capacity	√
121	WJ-MSCs	TNF- α and IFN-γ (Inflammatory cytokines, 50ng/ml/each for 48 h)	Immunosuppressive effects on mDCs and T cells isolated from patients with T1D	X
127	UC-MSCs (diabetic)	Metformin (1 mM), Lactoferrin (500 µg/mL) or TUDCA (2 µM) for 24 h	Restored proliferation and migration capacity and Inhibited cell stress	X
142	UC-MSCs	3D culture	CM from 3D cultured MSCs induced the Treg population and regulated cytokine release in T1D model	√
128	UC-MSCs	Melatonin (10 µΜ for 24 h)	Anti-T2D potentials (hypoglycemic effect, anti-IR, islet recover, regulating hepatic glucose metabolism)	√
137	UC-MSCs	GE (overexpress Exenatide, GLP1 analogue)	Beta-cell regenerative capacity	√
138	UC-MSCs	GE (overexpress IL-10, antinflammatory mediator)	Anti-inflammatory and anti-obesity potentials in DIO	√
123	ASCs (From healthy or non-obese T2D patients)	IFN-γ TTT (10ng/ml for 48 h)	Immunomodulatory Functions	X
24	ASCs (From healthy or obese T2D patients)	IFN-γ TTT (10ng/ml for 48 h)	Immunomodulatory Functions	X
139	UC-MSCs	GE (overexpress IFN-γ-inducible CXCL11 synthetic promoter)	Anti-inflammatory response	X
130	ASCs (diabetic)	DFX TTT (a hypoxia mimetic agent, 300 µM for 24 h)	Angiogenic capacity	X
33	UC-MSCs	DFX TTT (150 µM)	Immunomodulatory Functions in T1D	√
143	BMSCs	3D culture priming	T cell immunosuppressive potential	X

*ASCs* Adipose tissue-derived mesenchymal stem/stromal cells (MSCs), *BM-MSCs* Bone marrow-derived MSCs, *Cat* Catalase, *CXCL11* C-X-C Motif Chemokine Ligand 11, *CPT1A* Carnitine palmitoyltransferase 1 A, *DFX* Deferoxamine, *DIO* Diet-induced obesity, *DPSCs* Dental pulp stem cells, *GE* Genetic Engineering, *GLP1* Glucagon-like-peptide 1, *h* Hour, *hMSCs* human MSCs, *HF-MSCs* Hair follicle-derived MSCs, *HO-1* Heme oxygenase 1, *IFN-γ* Interferon gamma, *IL-10* Interleukin 10, *IR* Insulin resistance, *mDCs* mature dendritic cells, *SDF-1α* Stromal-derived factor 1 alpha, *sTNF-αR* soluble tumor necrosis factor-ɑ receptor type I, *SOD-2* Superoxide dismutase 2, *SIRT1* Sirtuin 1, *siRNA* Small interference ribonucleic acid, *T1D* Type 1 diabetes, *T2D* Type 2 diabetes, *TTT* Treatment, *TIMP-1* Tissue inhibitor metalloproteinase 1, *TNF-α* Tumor necrosis factor alpha, TUDCA (Sodium tauroursodeoxycholate), *UC-MSCs* Umbilical cord-derived MSCs, *WJ-MSCs* Wharton’s Jelly-MSCs

In vitro, a mixture of IFN-γ and TNF-α boosted the hWJ-MSC modulation of the profiles and functions of mature DCs and activated T cells that were differentiated from T1D patients [122]. IFN-γ-induced IDO expression may underly the strong immunosuppressive effect of inflammatory primed MSCs [24]. Bench studies indicate that preconditioning with deferoxamine (DFX, a hypoxia mimetic agent) could enhance the MSC regenerative secretome [33]. DFX treatment was able to restore the angiogenic potential of hASCs isolated from patients with T2D via hypoxia inducible factor 1 α (HIF-1 α)-dependent mechanism [130]. Resveratrol, a potent antioxidant, has been documented to mitigate TNF- induced inflammation in hBMSCs [125] or hDPSCs [126] via upregulating Sirtuin 1 expression or activation autophagy to inhibit (JNK) MAPK, respectively. A recent comprehensive review summarizes pharmacological agents that could promote the therapeutic efficacy of MSC transplantation in diabetes, with a focus on correcting the mitochondrial dysfunction of diabetic MSCs for autologous implications [129].

In vivo, substantial improvements in immunomodulation and beta-cell regeneration in STZ-T1D model were seen with DFX-preconditioned hUC-MSC-derived conditioned medium (CM) [33]. Metformin, insulin sensitizing drug, potentiated the therapeutic efficacy of hASCs in HFD-diabetic mice as shown by enhanced reversal of hyperglycemia, hyperinsulinemia and triglyceridemia [99]. Metformin was also able to counteract high glucose-induced cell stress in hUC-MSCs, as represented by the significant decrease in the transcriptional levels of senescence, proinflammation, ER stress markers [127]. As well, melatonin treatment effectively potentiated the hypoglycemic effects of hUC-MSCs in T2D model via potent PI3K/AKT-amelioration of IR and regulation of hepatic glucose metabolism. RNA-seq analysis revealed significant differential expression of genes that enrich cell proliferation and migration in melatonin-primed UC-MSCs [128]. The aggregation of MSCs into 3D spheroids could act as a functionalized formulation, supporting the administration of MSC spheroids for a sustainably improved immunosuppressive potency. In T1D model, the superior immunosuppressive capacity of CM-harvested from 3D over that from 2D- cultured UC-MSCs was evidenced, and augmented IL-4 release by the 3D formulation was suggested as an underlying mechanism [142]. Next generation sequencing revealed differential immune-modulation gene expression signatures between 3D cultured and the pro-inflammatory factor treated-MSCs indicating distinct immunosuppressive mechanisms engaged by the different priming strategies [143].

Numerous reports indicated that genetic modification of hMSCs could improve their experimental anti-diabetic efficacy [131–139]. hBMSC overexpressing the angiogenic factor VEGF, exhibited a sustained potential to reverse hyperglycemia in diabetic mice which was correlated with the activation of insulin/insulin-like growth factor (IGF) receptor signaling pathway involved in maintaining beta-cell mass and function [131]. Interestingly, hair follicle-derived MSCs (HF-MSCs) were engineered to overexpress human insulin gene and release human insulin in a time-and dose-dependent manner in response to rapamycin. When mice with STZ-T1D were engrafted with those engineered HF-MSCs, the cells expressed and released a dose of human insulin, dramatically reversed hyperglycemia, and significantly reduced death rate [132]. Betatrophin-transduced hASCs exhibited a stronger islet-supportive ability and a better therapeutic efficacy in STZ-T1D model than non-engineered ones [133]. Betatrophin is a hormone that can increase the production and expansion of insulin-secreting beta-cells when administered to mice [144]. Gao et al. [134]. reported that WJ-MSCs over expressing the newly identified adipokine, apelin, could provide a promising therapeutic option for management of T2D at clinical level. In that study, T2D rats infused with WJ-MSCs-apelin significantly decreased BGL by two weeks post-infusion. Transplantation of WJ-MSCs-apelin not only improved significantly insulin sensitivity and glucose disposal, but also promoted endogenous pancreatic beta-cell proliferation (9.6-fold increase compared to the control group). The inflammatory cytokines IL-6 and TNF-α were significantly decreased, whereas anti-inflammatory factor adiponectin was significantly increased after WJ-MSC-apelin injection. In another study, intraperitoneal administration of anti-oxidant modified hASCs, overexpressing *SOD2* (superoxide dismutase 2 gene) or *Cat* (catalase gene), in HFD-diabetic mice improved glucose tolerance and reduced systemic inflammation and fatty liver [135]. Wang et al. [137]. transduced UC-MSCs to overexpress exenatide, GLP1 analogue, and compared their beta-cell regenerative ability in NOD mice with non-transduced UC-MSCs. Exenatide-UC-MSCs exhibited superior anti-T1D potential (repressing insulitis and promoting beta-cell regeneration and insulin production). Bioinformatic studies predict that the effects of exenatide-UC-MSCs correlate with decreased abundance of pro-inflammatory intestinal bacteria and increased abundance of anti-inflammatory intestinal bacteria. An interesting Germany research designed and transduced hUC-MSCs with a synthetic inflammation-inducible promoter (CXCL11 promoter) to conditionally overexpress IL-10, which potentiate MSC therapy in inflammation-driven diseases [139].

In the context of PIT, hASCs preconditioned with a mixture of hyaluronic, butyric, and retinoic acids exhibited a superior potential to support the vascularization and function of an islet graft in diabetic rats, compared with naïve hASCs [66]. hASCs exposed to the mixture were able to increase the secretion of VEGF, as well as, the expression of angiogenic genes, including VEGF, kinase insert domain receptor (KDR), and HGF. That study suggests a novel strategy of MSC preconditioning to remarkably improve the efficacy of islet-hMSC cotransplantation [66]. Genetically modified hMSCs to overexpress soluble tumor necrosis factor-ɑ receptor type I (sTNF-αR) and heme oxygenase (HO)-1 genes (HO-1/sTNF-αR) exhibited improved survival of porcine islets and could reverse hyperglycemia more than porcine islets not treated with MSCs or islets cotransplanted with naïve/non-modified MSCs [140]. The present findings support the combined gene and MSC therapy for DM management. However, sufficient data for the clinical proficiency of primed hMSCs either with small molecules or biomolecules or genetic engineering is still required [141].

### Clinical outcomes: evaluation of hMSC-based therapy in patients with T1D or T2D

In the preclinical investigations, hMSCs have shown outstanding outcomes in treating T1D and T2D animal models. Administration of purified hMSCs, from various sources has also been considered clinically safe and effective for diabetic patients [8, 30, 32, 85, 145–160] (Table 4) and the therapeutic outcomes and safety concerns are summarized in supplemental Table 1.

**Table 4:** MSC-based therapy clinical studies in patients with T1D or T2D

Ref	Country	Study Type	MSC source	DM type	MSC-treated patient Size	Patients Age range (Y) Mean or range	BMI (kg/m^2^)	Duration of the disease (Y or M) Mean or range	Cell Dose and Frequency of Regimen	Allogenic vs. Autologous	Route Of Administration	Follow-up Duration (M)
145	China	Pilot study	PL	T2D	10	30–85	ND	≥ 3 Y	Three doses/one-month interval The total cell no. /pt with an average of 1.35 × 10^6^ cells /kg	Allogenic	IV	6
30	China	RC, double-blinded	WJ	T1D	15	17.6 ± 8.7	20.9 ± 3.7	Newly onset, however, the exact duration was ND	Two doses (4 week-interval) Total mean cell number was 2.6 ± 1.2 × 10^7^	Allogeneic	IV	24
146	China	single-center prospective study	WJ	T2D	22	52.9 ± 10.5 (18–70)	25.1 ± 2.4	8.7 ± 4.3Y	Allogenic	Two doses (5-days interval) (1 × 10^6^ cells/kg/dose)	IV & IP endovascular	12
147	Sweden	Single-center, RC, Open-label, pilot study	BM	T1D	9	Mean: 24 ± 2 (18–40)	23.3 ± 1.1	Diagnosed < 3weeks before enrollment	Autologous	2.1–3.6 × 10^6^ cells/kg (median 2.75 × 10^6^ cells/kg)	IV	12
148	China	Pilot study	UC	T2D	6	40.5± 3.76	23.7 ± 0.29	42.7± 13.02 M	Allogenic	1 × 10^6^ cells/kg BW (two doses with a two-week interval)	IV	24–44 [33.2± 2.82]
160	China	RC, open-label	UC	T1D	21	18–40	22.06 ± 2.46	$\:\ge\:$2-$\:\le\:$16	Allogenic MSCs and autologous BM-MNC	Single dose 1.1 × 10^6^/kg UC-MSC & 106.8 × 10^6^/kg aBM-MNC	DPA or its subsitute	12
149	China	RC, double-blinded	WJ	T2D	31	52.43 ± 4.88	26.74 ± 5.41	8.93± 5.67 Y	Twice 4-weeks interval Mean cell number was 6.1± 2.1 × 10^7^	Allogenic	IV	36
32	China	cohort study	BM	T1D	5	14–31	15.8–20.1	3.5–11 M	Allogenic	A single dose (1 × 10^6^ cells/kg BW)	IV	48
150	India	RC	BMSC vs. BM-MNC	T2D	19	30–60	28.1	≥ 5 years	Autologous	1 × 10^6^ cells/kg BW for aBMSCs 1 × 10^9^ cells/patient aBM-MNC	SPD	12
151	Kazakh-stan	prospective cohort study	BM	T1D	5	20–42	ND	ND	Autologous	95–97 × 10^6^	IV	3
152	Brazil	Prospective, RC, single-center, open trial, phase II,	AT	T1D	8	16–35	20.76–26.06	< 4 M	Allogenic	ASCs: 1 × 10^6^ cells/kg BW (Single dose) & cholecalciferol 2000 UI/day for 3 months	IV	3
153	China	Pilot	Deciduous teeth	T2D	22	55.96 ± 4.81	24.42 ± 2.64	> 5Y	Allogenic	SHED: 1 × 10^6^ cells/kg BW (Three doses at two week- interval	IV	12
155	Iran	R, non-C, open label phase 1 clinical trial	PL	T1D	4	12–18	15.4–22	< 6 M	Allogenic	ASCs: 1 × 10^6^ cells/kg BW (single dose)	IV	12
156	Vietnam	R, non-C, open-label	BM	T2D	30	55–66	40% <23 60% >23	46% ≤10 Y 54% >10 Y	Autologous	Single dose 1 × 10^6^ cells/kg BW	IV (*n* = 15) or DPA (*n* = 15)	6 (*n* = 29) 12(*n* = 25)
157	Iran	R, PC, double-blinded	BM	T1D	11	10.27± 1.67	16.75 ± 2.57	**Early group** received MSC therapy *during the first year* of diagnosis & **late group** received the treatment *1-year after diagnosis*	Autologous	Two doses (1 × 10^6^ cells/kg BW dose) Three week-interval	IV	12 For each group
158	China	Pilot study	UC	T2D	16	52.5 ± 7.91	24.47 ± 2.76	10.06 ± 5.74 Y	Allogeneic	Three doses (1 × 10^6^ cells/kg BW dose) Once weekly	IV	≈ 3
159	China	A single-center, R, PC, double- blinded	UC	T2D	45	50.00± 9.38	28.69 ± 3.35	11.44± 4.78 Y	Allogenic	Three doses with one- month interval (1 × 10^6^/ Kg BW/ dose)	Elbow joint (IV)	12
85	China	RC, OPEN TRIAL	BM or UC	T1D	14 BMSCs (*n* = 4) UC-MSCs (*n* = 10)	15 (Median)	17.3 ± 2.0	1 M (MEDIAN)	Allogenic	Single dose 1 × 10^6^ cells/kg body	IV	12

*AT* Adipose tissue, *ASCs* adipose tissue-derived mesenchymal stem/stromal cells, *BMI* Body mass index, *BM* Bone marrow, *BM-MNC* Bone marrow mononuclear cells, *BW* Body weight, *C* Controlled, *DPA* Dorsal pancreatic artery, *Gp* Group, *IV* intravenous, *IP* Intrapancreatic, *KA* Ketoacidosis, *M* Months, *MSC* Mesenchymal stem/stromal cells, *ND* not-defined, *PC* Placebo controlled, *PL* Placenta, *R* Randomized, *RC* Randomized controlled, *SPD* Superior pancreatico-duodenal artery, *T1D* type 1 diabetes, *T2D* type 2 diabetes, *TTT* Treatment, *UC* Umbilical Cord, *WJ* Wharton’s jelly, *Y* Years

In the context of T1D, the effectiveness of autologous BMSCs (aBMSCs) was tested [147, 151, 157]. aBMSCs administered IV to patients with newly-onset T1D significantly improved the C-peptide response in a mixed-meal tolerance test during the first-year post-therapy, indicating intervening in the disease process and preserving the beta-cell function [147]. Furthermore, IV transplanted aBMSCs, in another cohort of patients with T1D showed, from the first month, a decrease in the doses of daily insulin, while it caused relatively small change in glycated hemoglobin (HbA1c) and leptin level. By the third month, they enhanced a significant increase in the leptin level [151]. Izadi et al. [157]. addressed the therapeutic effect of IV injection of two doses of aBMSCs in children with T1D (early/ during the first year of diagnosis vs. late/ one year -post diagnosis). The factor of exercise and patient life style was also considered and the patients were followed for at least one year-post transplantation. Despite the non-decrease in exogenous insulin dose, the therapy achieved efficacy by normalizing HbA1c and controlling immunological responses in the patients (decreased serum TNF-α, increased serum IL-4 andTregs frequency in the peripheral blood). Early MSC transplantation offered advantages over the late one as it caused higher reduction in HbA1c and the serum TNF- α, however, a significantly higher increase of C-peptide and the serum levels of TGF-β1, IL-10, and IL-4. Noteworthy, exercise enhanced MSC transplantation efficacy in early and late groups for the favor of improved quality of life and better metabolic indices [157].

Allogeneic MSCs isolated from adult [32, 85, 152], or extraembryonic [30, 85, 155, 160], sources have also shown promising outcomes in patients with different T1D diagnostic onset. WJ-MSCs were induced a significant reduction of postprandial plasma glucose (PPG) & HbA1c, however, a significant increase of fasting C-peptide (FCP) over 24 months follow-up in fifteen patients with newly onset-T1D [30]. Fluctuated insulin intake after therapy was reported, 20% of patients suspended exogenous insulin, around 53% and 6% of patients reduced the dose by > 50% and 15–50%, respectively. Li and his colleagues [32] recruited a total of five T1D patients with ketoacidosis and treated them with allogenic hBMSCs. Mean daily exogenous insulin dosages required to control hyperglycemia individually were recorded and the levels of fasting and postprandial plasma C-peptide (PCP), as well as, HbA1c over a 4 years follow-up period were determined longitudinally. Patients responded differentially to MSC therapy, however, 80% of patients were responders. That was represented by (1) lower levels of HbA1c, as compared with that before treatment, for at least three years, an indication of effective control of hyperglycemia, and (2) slower decrease of FCP or PCP, indicating preserved beta-cell functions. Additionally, 60% of patients reduced mean daily insulin dosage for at least two years by > 40%. Cai et al. [160]. transplanted combined WJ-MSC and auologus bone marrow-mononuclear cells (aBM-MNCs) in patients with established T1D through supraselective pancreatic artery cannulation with follow up for one year (3 months intervals). The authors reported significant improvements in FCP, C-peptide area under curve (AUC_C−Pep_), and insulin area under the curve (AUC_Ins_) during oral glucose tolerance test, at 1-year post-therapy, as evidences of enhanced insulin production and reduced insulin need. A small change/increase in the FCP after therapy was considered significant, taking into account the negligible or non-existing basal level of FCP by long disease incidence. In addition, compared to baseline and the control group, HbA1c, and fasting blood glucose (FBG) were reduced. Furthermore, serum levels of Th1 cytokine, IFN-γ, and ATP generation by CD4^+^T cells were decreased after therapy, suggesting T-cell inactivation. Additionally, patient serum levels of the regulatory cytokine IL-10 were elevated [160]. Another report evaluated the short-term efficacy of a combined therapy of allogenic hASCs and calciferol in patients with newly diagnosed T1D (< 3 months). At the end of the observation period, significantly lower insulin doses and HbA1c in the treatment group, as compared with the control standard insulin treated group, were reported. However, c-peptide did not differ between the treatment and the control groups. The glycemia control-mediated effect of the combined therapy was likely attributed to the significant upregulation of the frequency of the immunomodulatory CD8^+^ FOXP3^+^ Tregs post-treatment. A larger sample and a longer follow-up period are necessary to further determine the safety of the treatment and the efficacy of ASCs infusion combined to Vitamin D supplementation for recent-onset T1D [152]. The short-term efficacy of PL-MSCs in newly-onset juvenile T1D has been clarified in four patients. PL-MSC injection decreased specific and sensitive antibodies in T1D pathogenesis (ZnT8-Ab and anti-Gad-Ab) till month 3 of follow up, then they increased again [155]. The effect of MSC source on the efficacy of MSC therapy in diabetes was evaluated. Allogeneic BMSCs or WJ-MSCs were administered through IV in patients with T1D who were observed for 12 months. WJ-MSCs showed advantages over BMSCs as they induced a greater reduction of HbA1c % and better improvement of FCP [85].

In the context of T2D, few studies were found to address the therapeutic efficacy of aBMSCs [150, 156]. Bhansali et al. [150]. compared the efficacy of aBMSCs vs. aBM-MNCs by administering them through the superior pancreatico-duodenal artery in patients with established T2D (≥ 5yrs disease diagnosis), and changes in metabolic indices were observed over the course of one year. aBMSCs and aBM-MNC demonstrated a significant reduction in insulin requirements (≥ 50% from baseline). Specifically, aBMSCs increased the expression of the IRS-1 gene, resulting in enhanced insulin sensitivity, whereas, aBM-MNCs improved glucagon-stimulated C-peptide response during hyperglycemic clam, providing newer insights in T2D cell-based therapy. In a critical clinical trial conducted by Nguyen and his colleagues [156] BMSCs were administered into patients with T2D disease duration ≤ 10 years vs. those with a disease diagnostic onset > 10 years and BMI < 23 vs. > 23 kg/m^2^ via IV or IP (dorsal pancreatic artery/DPA) with follow-up for almost one year and three months-time points. It was illustrated that the route of administration didn’t affect the efficacy of aBMSC therapy, however, it was greatly correlated with the disease duration and patient’s BMI. Patients with T2D duration ≤ 10 years and BMI < 23 kg/m^2^ showed significant reduction in both HbA1c and remarkable decrease of FBG with diminishing effectiveness over the time (short-term efficacy). Interestingly neither duration nor BMI affected C-peptide level which showed same change in all treated groups. Insightful investigation revealed that T2D duration badly affected the proliferation rate, abrogated the glycolysis and mitochondria respiration of BMSCs, and induced the accumulation of mitochondria DNA mutation in BMSCs, explaining the loss of efficacy of 10-years or more diabetic BMSCs.

Diverse investigators assessed the efficacy and safety of allogeneic MSCs derived from adult [153], or perinatal [145, 146, 148, 149, 158, 159], tissues in T2D patients illustrating promising results. Li et al. [153], assessed the therapeutic efficacy of SHED transplantation in patients with T2D > 5 years and one-year follow up. The SHED effectively improved metabolic glucose and lipid indices. Analysis revealed that the patient BGL before SHED therapy was correlated with the efficacy, where patients with HbA1c < 8.5 and total cholesterol < 5 mmol/L or triglyceride ≤ 1.5 mmol/L or low-density lipoprotein cholesterol < 3.2 mmol/L reduced significantly the daily insulin dose. The islet function state of the patients before treatment was closely related to the degree of islet function recovery after treatment, such that patients with FCP 1.7 ng/mL and PCP at 2 h > 3 ng/mL showed better islet function recovery after treatment. Such findings support blood lipid levels and baseline islet function may serve as key factors contributing to the therapeutic outcome of MSC transplantation in patients with T2D. Jiang et al. [145]. investigated the efficacy and safety of IV administration of PL-MSCs in patients with established T2D. Six months-post therapy, significant reduction in the mean daily insulin dosage and HbA1c %, while, significant increase in c-peptide, were detected. Additionally, the renal and cardiac functions were improved and no adverse reactions were recorded [145]. Furthermore, WJ-MSCs were tested for their long-term effects by administering them intravenously to T2D patients and following them for 3 years. PPG and HbA1c levels significantly decreased after treatment, accompanied by a significant increase in FCP [30]. Non-significant decrease in homeostatic model assessment of IR (HOMA-IR), IR indices, was detected in the first year, followed by re-increase. In that study, the differential effect of WJ-MSC on dosages of daily insulin and oral hypoglycemic drugs was clarified and 32.3% of patients remained insulin free for 12.5 ± 6.8months. Moreover, compared with the control group/sham-treated, WJ-MSC infusion decreased significantly the incidence of diabetes complications [30]. In accordance, Liu et al. [146]. stated that transplantation of two doses of WJ-MSCs in patients with T2D via IV, then IP endovascular routes regulated significantly, at 1-year, the PPG, FCP, and beta-cell function [represented by homeostatic model assessment of beta-cell (HOMA-β)]. In terms of insulin requirements, 94% of patients who were receiving insulin, exhibited differential decline in insulin dosage post transplantation and 41% of insulin-dependent patients suspended insulin for 9 months. Additionally, immunological tests revealed a decrease in the counts of CD3, CD4, and CD8 lymphocytes, with significant changes for CD3 and CD4 T lymphocytes at 6 months post-transplant. At the same time point, a significant decrease in the serum inflammatory markers, IL-6 and IL-1β was also detected. The correlations between the change in the levels of FCP and the counts of CD3^+^ T lymphocytes and serum IL-6 level were significant. Such findings confirm that MSC anti-diabetic effect in clinic is mediated at least partially via modulating inflammation [146]. In a pilot study, hUC-MSCs were transplanted intravenously into six patients with T2D, who were then monitored for more than 24 months. 50% of patients became insulin-free for the whole follow-up period, while the remaining three patients reduced their insulin demands. In the insulin-free group, post MSC therapy, levels of FCP and c-peptide release in response to meal increased significantly within one month and remained high during the followup period. Additionally, HbA1c significantly reduced with a stable level over the 24-month time. In contrast, the insulin-dependent group, post MSC therapy, showed a significant reduction in HbA1c for only 3 months and did not exhibit any significant change in C-peptide levels [148]. In a preliminary short-term evaluation (≈ 3 moths) of the efficacy and safety of hUC-MSCs in patients with T2D and a mean disease duration 10.06 years, the authors reported that hUC-MSCs could ameliorate hyperglycemia by decreasing FBG and HbA1c and reducing the dosage of hypoglycemic agents. It also improved islet beta-cell function. However, no significant improvement of IR and no significant decrease in FCP and PCP during the follow-up period was reported [158]. The safety and effectiveness of hUC-MSCs were also evaluated in Chinese people suffering from T2D. The UC-MSCs were administered IV three times (one dose /month) at the elbow joint and the patients were investigated for one year. The treatment resulted in significant decrease in daily insulin requirement and HbA1c levels, and ameliorating IR, as represented by improved glucose infusion rate, in a time-dependent manner. Only 20% of patients achieved the study goal (HbA1c levels < 7.0% and daily insulin reduction of ≥ 50% at the end of follow-up [159]. In conclusion, the above-mentioned studies recommend the effectiveness of MSCs in controlling metabolic indices in T1D or T2D patients. Additionally, enhanced physical and mental quality of life measures were observed after MSC transplantation [157, 160]. Importantly, MSC injection was associated in some reports with transient easily-resolved adverse events such as abdominal pain, fever, fatigue, headache, vomiting or bleeding at site of injection [153, 156, 158, 160]. MSC transplantation significantly reduced the incidence of hypoglycemic episodes, relative to standard insulin treatment, suggesting the safety of MSC-based therapy in DM [153, 157, 160].

### MSC-based therapy in DM perspectives and limitations

Accumulating experimental and clinical data indicate that MSCs from adult or perinatal tissues serve as ideal candidates for the treatment of DM due to their great advantages in terms of abundance, high proliferative phenotype, immunomodulation and plasticity for IPC generation [27]. The UC and its main component WJ are normally discarded after a birth and poses no risk for collection. Importantly, hUC- and hWJ- MSCs have been widely used in DM cell-based therapies at the pre-clinical and clinical levels [161]. However, the efficient translation of the routine application of these cells in DM cure, large highly-standardized clinical trials can be planed. Such trials should be unified in sample inclusion and exclusion criteria, disease duration/stage, sample size, and investigated metabolic parameters for continuous follow-up. Importantly, banking of these cells (autologous or allogenic) needs special attention.

From clinical perspectives and depending on the available data, many uncertainties need deeper research to draw a possible effective therapeutic regimen for MSCs or their derivatives (EVs or EX) for DM in clinics. Among the variables of future research interest is the optimal MSC source to treat DM. Equivalent effectiveness, of UC-MSCs and BMSCs in glycemic control and beta-cell preservation at both the preclinical and clinical levels, has been reported [85]. In another report, the same donor-derived AM-MSCs and UC-MSCs possessed comparable effects and shared a similar hepatoprotective mechanism on the alleviation of experimental T2D symptoms [48]. Controversially, Ma et al. [39], reported that MSC types exhibit differential potential to ameliorate preclinical T2D, UC-MSCs presented super anti-hyperglycemic, anti-IR, and anti-hyperlipidemia effects over DPSCs and ASCs, however, ASCs showed the strongest liver lipogenesis inhibition. The optimal MSC tissue source for efficient MSC therapy in DM could thus depend on the detailed biochemical and histopathological examination of the diabetic patients, not only broad categorization as patients with T1D or T2D. The findings may also support the therapy by MSCs pooled from different sources to cover the diverse disease pathological mechanisms. As well, among the challenges that face the choosing of the optimal MSC source and the effective clinical translation of MSCs is their inherent heterogeneity which complicates the safety and consistency of the therapeutic outcomes [162]. The advent of single-cell RNA-seq (scRNA-seq) has enabled precise MSC characterization and biomarker identification, revealing the diversity of MSC subclusters and their specific transcriptome patterns and functions [163]. scRNA-seq and developmental trajectory analysis of MSCs derived from different human sources may identify subpopulations with superior therapeutic properties, particularly in DM. This in-depth knowledge is crucial for the optimal MSC source selection, targeted MSC-based therapies development and clinical applications refinement].

Despite the encouraging experimental results, the duration of efficacy of a single MSC infusion is relatively transient [36, 89]. Si et al. [89]. found that the antidiabetic effect of a single MSC infusion was maintained for less than 4 weeks in T2D rats. Clinical trials also exhibited similar results [30, 146]. To overcome such limitation, repeated MSC administrations may prolong the anti-diabetic effect. Alternatively, the combined administration of MSCs with a pharmacological agent, an epigenetic modifier [36, 92], or hyperbaric oxygen therapy [93], to augment MSC immunomodulatory and anti-inflammatory responses and sustain their anti-diabetic effects, can be introduced in clinics. Moreover, the infusion of MSCs to diabetic patients follow fasting- mimicking diet (FMD) regimen can be applied to achieve a better improvement in restoring lipid metabolism, as reported at the preclinical level in mice with T2D [95]. FMD is a kind of caloric restriction which represents a dietary mode low in calories, sugars, and proteins but high in unsaturated fats, can dramatically reduce triglycerides and total and low-density lipoprotein cholesterol, resulting in a loss of total body fat and a reduction of liver fat accumulation [164]. Patient life style, nutrition, exercise, and microenvironment could impact the MSC therapeutic outcome [157] and that can be uniformly considered in the future clinical studies.

Disease duration greatly influence the therapeutic efficacy of MSCs and their functional characteristics, affecting the autologous implications [156, 157]. Similar results were reproducible at preclinical level, where the efficacy of UC-MSCs to reverse beta-cell dedifferentiation in T2D model was reduced in the late-stage treatment, relative to the early stage one [62]. Thus, the precise selection of patients who may benefit from MSC treatment, depending on the onset of diagnosis and the disease stage, is really crucial from a clinical standpoint [116]. Most of the reported clinical studies (≥ 95%) injected MSCs intravenously in patients with T1D or T2D. However, those studies have not tacked MSCs in vivo and they have not considered the potential lung trapping [165]. Few studies explored the efficacy of DPA administration [156, 160] or intraportal infusion [166]. Further comparative clinical studies in the context of MSC delivery route are therefore implored. To optimize MSC tracking [167], preclinical research, focusing mainly on comparing different routes at the same set-up, to elaborate knowledge about the route-MSC pancreatic homing efficacy and therapeutic outcome, are potentially encouraged.

Different MSC priming approaches, reviewed here, were only performed on bench or in diabetic animal models. In order to accelerate the translation of the innovative MSC enhancement strategies into the DM clinics, several key issues have been previously introduced by Li et al. [120], including: (1) setting-up a quality control strategy for manufacturing clinical grade primed or genetically modified MSCs, (2) establishing an efficient screening system to exclude primed cells with oncogenic mutations and, (3) precise patient selection to enroll patients who most likely derive maximal benefits from those strategies.

In the context of MSC-secretome and EVs, almost no clinical translation in DM has been reported yet, even MSC secretome is a factor-rich protein-based biotechnological product with a greater safety when compared to administration of living human cells, so presenting virtually no/low risk [161]. Thus, preliminary clinical trials evaluating the efficacy and safety (immunogenicity and tumorgenicity) of primed MSCs, with different augmenting approaches, and MSC-derived EVs or secretome in precisely selected diabetic patients are recommended.

## Conclusion

In summary, the clinical studies demonstrated a potential benefit of MSC administration for the treatment of T1D (especially the early onset) and T2D, however, considerable number of critics remain not-fully explored and a final conclusion cannot be drawn. The methodological aspects of the identified studies and findings are heterogeneous, challenging the interpretation of the actual DM-MSC therapeutic impacts and methodically rigorous research is further needed to increase credibility. Thus, high-quality, large-scale randomized clinical studies are demanded to provide a definitive conclusion. At the preclinical level, standardized research in non-murine large diabetic animal models, considering the genetic defect(s), may decrease the translation gap between the murine models and human patients in hMSC-based DM therapy.

## Electronic supplementary material

Below is the link to the electronic supplementary material.

Supplementary Material 1

## Data Availability

Non-applicable.
