# Supplementary material for: Human mesenchymal stem/stromal cell based-therapy in diabetes mellitus: experimental and clinical perspectives
Source: Stem Cell Res Ther. 2024 Oct 29;15:384. doi: 10.1186/s13287-024-03974-z (PMC11520428; doi:10.1186/s13287-024-03974-z)
Supplement: Supplementary file 1 — Supplementary Material 1 [file 13287_2024_3974_MOESM1_ESM.docx]

**Supplemental Table 1: Outcomes and Safety of MSC-based Therapy Clinical Studies in patients with T1D or T2D**

| **Ref** | **DM type** | **BMI**  **(kg/m^2^)** | **Duration of the disease**  **(Y or M)**  **Mean or range** | **Cell Dose and Frequency of Regimen** | **Route**  **Of Administration** | **Follow-up**  **Duration**  **(M)** | **Significant metabolic outcomes** | **Safety** |
| --- | --- | --- | --- | --- | --- | --- | --- | --- |
| **145** | T2D | ND | ≥3 Y | Three doses/one-month interval  The total cell no. /pt with an average of  1.35x 10^6^ cells /kg | IV | 6 | ***-Reduced daily insulin dose*** [from 63.7 ± 18.7 to 34.7 ±13.4 IU, P < 0.01]  ***-Increased C-peptide level*** [from 4.1 ± 3.7 ng/mL to 5.6 ± 3.8 ng/mL, P < 0.05]  **-Decreased HbA1c** (from 9.8±2.2 to 6.7±1.2, P < 0.01] | No AEs |
| **30** | T1D | 20.9±3.7 | Newly onset, however, the exact duration was ND | Two doses  (4 week-interval)  Total mean cell number was 2.6 ± 1.2 × 10^7^ | IV | 24 | Relative to the control group,  MSC induced:  Significant ***reduction of PPG & HbA1c***, however, ***significant increase of FCP and CPGR*** (P<0.05).  -at the end of follow-up, ***insulin dose reduction***:  20% of responders discontinued insulin  53% reduced insulin dose by >50%, & 6.6% by 15-50%. | NO AEs |
| **146** | T2D | 25.1 ± 2.4 | 8.7 ± 4.3Y | allogenic | IV &  IP endovascular | 12 | **% Of responder patients**: all patients responded with different degrees.  At the 12th month,  **HbA1c decreased** from (8.20 ± 1.69% to 7.0 ± 0.60%, P<0.01)  ***PPG* decreased (**14.96 ± 4.54 mmol/L to 12.25 ± 3.83 mmol/L, P<0.01)  **FCP increased** from (1.29 ± 0.83 ng/mL to 1.86 ± 1.0 ng/ml, P<0.05)  **HOMA-β increased** from 65.99 ± 23.49% to 86.0 ± 37.1% (***indication for improved beta-cell function***)  -**Reduction of baseline treatment** (29% of insulin-dependent patients and 80% of OHD-dependent reduced their respective therapy by > 50% | Mild post-operative side reactions, which rapidly recovered |
| **147** | T1D | 23.3 ± 1.1 | Diagnosed <3weeks  before enrollment | autologous | IV | 12 | 67% of patient responded to treatment; they significantly ***increased delta values for both peak***  ***C-peptide response and AUC***  ***C-peptide response to the MMTT,*** | No |
| **148** | T2D | 23.7±0.29 | 42.7±  13.02 M | allogenic | IV | 24-44  [33.2±  2.82] | 50% of patients became insulin-free for 25-43M and the remaining three patients reduced the insulin dose, relative to the basal dose.  **Significant decrease of HbA1c**, P<0.01  **Significant increase of FCP and C-peptide release in response to mea**l. P< 0.05 | No adverse reactions reported |
| **160** | T1D | 22.06 ± 2.46 | $\geq$2-$\leq$16 | Allogenic MSCs and autologous BM-MNC | DPA or its subsitute | 12 | **% Of responder patients:95%**  Relative to control subjects, MSC gp:  **-Increased AUC_C-Pep_** by 105.7% (P = 0.00012)  **-Increased AUC_INS_** by 49.3% (P = 0.01)  **-Decreased HbA1c** by 12.6% (P < 0.01)  **-Decreased FBG** by 24.4% (P < 0.042)  **-Reduced daily insulin requirements** by 29.2% (P < 0.01) | transient abdominal pain, Bleeding at the puncture site and Upper respiratory tract infections were detected, however, they were resolved.  SCT significantly reduced the incidence of hypoglycemic episodes |
| **149** | T2D | 26.74±5.41 | 8.93±  5.67 Y | Twice  4-weeks interval  Mean cell number was 6.1±  2.1x10^7^ | IV | 36 | *Relative to the control gp, MSC gp exhibited*  ***-Significant reduction in HbA1c & PPG.***  -**Significant increase in FCP and HOMA-β**, indices for improved beta-cell function  -**Significant decrease of daily insulin and OHD**. | No |
| **32** | T1D | 15.8-20.1 | 3.5-11 M | allogenic | IV | 48 | **% Of responder patients:** 80%.  -Patients differentially responded in terms of ***exogenous insulin requirements*** (from being independent for a short-time to reducing the insulin dose by 50% for 1-2 years).  -In addition, ***slow reduction in FCP and PCP and decrease of HbA1c***. | NO |
| **150** | T2D | 28.1 | ≥5 years | autologous | SPD | 12 | 60% of patient are responders, both groups induced a significant reduction in insulin dose by ≥50% from baseline, at the 12 month.  aBM-MNC induced **a significant increase in 2nd phase C-peptide response** during hyperglycemic clamp  aBMSCs  induced a ***significant improvement in insulin sensitivity index*** | No |
| **151** | T1D | ND | ND | autologous | IV | 3 | After 1 month: decrease in daily insulin dosage levels, from 63 ± 8.83 to 50.2 ± 12.1 U (P = .064).  After 3 months: leptin levels increased from 6.86 to 10.77 ng/mL (P = .016). | No |
| **152** | T1D | 20.76-26.06 | < 4M | Allogenic | IV | 3 | **-Decrease in insulin requirements** (From 0.61±0.26 to 0.22 ± 0.17 IU/Kg; P = 0.01)  **-Decrease in HbA1c** (From 7.48 ± 0.52 to 6.47 ± 0.86 %; P = 0.03) | ND |
| **153** | T2D | 24.42  ± 2.64 | > 5Y | Allogenic | IV | 12 | At the end of the follow-up:  -86.36% of patients showed ***a significant reduction of glycosylated serum albumin level*** (P < .05) and 68.18% ***HbA1c*** (P < .05)  ***- Daily insulin reduced*** by 35.34% by the end of the treatment. The total effective rate was 68.18% (15/22) with three patients having ceased insulin injections.  ***A significant increase of the serum levels of PCP***, relative to the baseline (P < .05). | Hypoglycemia, with reduced frequency over time.  Some patients experienced a transient fever (11.11%), fatigue (4.17%), or rash (1.39%), which were easily resolved |
| **155** | T1D | 15.4-22 | < 6M | allogenic | IV | 12 | -***ZnT8-Ab decreased*** till month 3 and then increased again in all patients. Anti-***Gad-Ab decreased*** till month 3 of follow up then increased. | No serious adverse  reactions |
| **156** | T2D | 40% <23  60% >23 | 46% ≤10 Y  54% >10 Y | Autologous | IV (n=15) or  DPA (n=15) | 6 (n=29)  12(n=25) | Only, in MSC- group <10 years and <23 BMI, -***Significant Reduction of:***  ***- HBA1c*** from 8.9±1.6 to 8.2±0.5 (P=0.044)  -***FBG*** from 8.5±3.5 mmol/l to 6.8±2.4 mmol/l (P=0.133) | No severe AEs,  However,  . At the end of the trial, some transient AEs were observed including hyperglycemia (10%) and hypoglycemia 3%).  In addition to  feeling pain (10%), & single incidences of splenomegaly, insomnia, vomiting, headache, or hypertension |
| **157** | T1D | 16.75±2.57 | **Early group** received MSC therapy *during the first year* of diagnosis &  **late group** received the treatment *1-year after diagnosis* | autologous | IV | 12  For each group | Compared to placebo group, *MSC ttt induced* ***a significant reduction of HbA1c %*** at the 12^th^ month (P=0.043).  Compared to the late TTT group, early ttt showed**,**  -***A Significant decrease in HbA1c* %** at 12 months after transplantation (P=0.041)  **- *Higher serum c-peptide***  (P=0.016) at 9 months of follow-up | **MSC transplantation**  is safe and significantly reduced the number of hypoglycemic episodes |
| **158** | T2D | 24.47 ± 2.76 | 10.06 ±  5.74 Y | Allogeneic | IV | [≈ 3](https://wumbo.net/symbols/approximately/) | -***FPG was significantly reduced*** [from 9.34 to 6.52, P < 0.01] at 2 weeks.  -**The HbA1c level was significantly reduced** [from 7.8 to 7.15 P < 0.01, at 11 weeks.  -***The patients’ islet beta-cell function was significantly improved*** [from 29.9 to 40.97, P < 0.01] at 4 weeks.  -***The dosage of hypoglycemic agents was reduced in all patients***, of whom 6 (50%) had a decrement > 50% and 1 (6.25%) discontinued the hypoglycemic agents. | No serious AEs, however, transient fever or hypoglycemia was observed |
| **159** | T2D | 28.69±3.35 | 11.44±  4.78 Y | allogenic | Elbow joint (IV) | 12 | **% Of responder patients:** all patients responded, however only 20% achieved the study goal by the 12^th^ month (HbA1c % < 7% and daily insulin reduction by 50%)  -Relative to the placebo group, MSC-group exhibited  **-*Daily insulin intake reduction*** by 27.78%  **-*decrease in the levels of HbA1c*** by 1.31%  **-*Improved insulin resistance****,* as represented by a significant elevation in GIR (from 3.12 to 4.76 mg/min/kg, compared with from 3.26 to 3.6 in the placebo group). | **No** |
| **85** | T1D | 17.3±2.0 | 1M  (MEDIAN) | allogenic | IV | 12 | -All patients responded to MSC treatment and 28.6% of patients maintained clinical remission at the end of the follow-up.  -Compared to control, MSC therapy significantly ***reduced HbA1c*** and ***improved the decrease in FCP*** level by 25.1% & ***the PCP*** by 10%.  BMSCs group:  HBA1c decreased by 11.1% of the baseline level  UC-MSCs group:  HBA1c decreased by 25.1% of the baseline level  However, no significant differences between the two MSC groups in terms of other metabolic parameters improvementnt. | No major adverse reactions reported |

AEs: Adverse events, AT: Adipose tissue, ASCs: adipose tissue-derived mesenchymal stem/stromal cells, AUC_C-Pep_, C-peptide area under the curve, AUC_INS_: insulin area under the curve, BGL: Blood glucose level, BMI: Body mass index, BM: Bone marrow, BM-MNC: Bone marrow mononuclear cells, BW: Body weight, C: Controlled, CPGR: C-peptide/glucose ratio, DPA: Dorsal pancreatic artery, ICA: Islets cells antibody, FBG: fasting blood glucose, FCP: Fasting c peptide, GAD: Glutamic acid decarboxylase, Gp: Group, GIR: Glucose infusion rate, HbA1c, glycated hemoglobin, HBO: Hyperbaric oxygen, HOMA-β: Homeostatic model assessment of beta-cell, IV: intravenous, IP: Intrapancreatic, KA: Ketoacidosis, M: Months, MSC: Mesenchymal stem/stromal cells, MMTT: Mixed meal tolerance test, ND: not-defined, OHD: Oral hypoglycemic drug, PC: Placebo controlled, PL: Placenta, PPG: Post-prandial plasma glucose, PCP: Post-prandial c-peptide, R: Randomized, RC: Randomized controlled, SPD: Superior pancreatico-duodenal artery, T1D: type 1 diabetes, T2D: type 2 diabetes, TTT: Treatment, UC: Umbilical Cord, WJ: Wharton’s jelly, Y: Years, ZnT8-Ab: Zinc transporter 8 antibody.
